# An RGD-Modified MRI-Visible Polymeric Vector for Targeted siRNA Delivery to Hepatocellular Carcinoma in Nude Mice

**DOI:** 10.1371/journal.pone.0066416

**Published:** 2013-06-07

**Authors:** Chun Wu, Faming Gong, Pengfei Pang, Min Shen, Kangshun Zhu, Du Cheng, Zhihao Liu, Hong Shan

**Affiliations:** 1 Molecular Imaging Lab, Department of Radiology, The Third Affiliated Hospital of Sun Yat-sen University, Guangzhou, China; 2 Interventional Radiology Institute, Sun Yat-sen University, Guangzhou, China; 3 PCFM Lab of Ministry of Education, School of Chemistry and Chemical Engineering, Sun Yat-sen University, Guangzhou, China; 4 Molecular Digestive Lab, Department of Gastroenterology, The Third Affiliated Hospital of Sun Yat-sen University, Guangzhou, China; Icahn School of Medicine at Mount Sinai, United States of America

## Abstract

RNA interference (RNAi) has significant therapeutic promise for the genetic treatment of hepatocellular carcinoma (HCC). Targeted vectors are able to deliver small interfering RNA (siRNA) into HCC cells with high transfection efficiency and stability. The tripeptide arginine glycine aspartic acid (RGD)-modified non-viral vector, polyethylene glycol-grafted polyethylenimine functionalized with superparamagnetic iron oxide nanoparticles (RGD-PEG-*g*-PEI-SPION), was constructed as a magnetic resonance imaging (MRI)-visible nanocarrier for the delivery of Survivin siRNA targeting the human HCC cell line Bel-7402. The biophysical characterization of the RGD-PEG-*g*-PEI-SPION was performed. The RGD-modified complexes exhibited a higher transfection efficiency in transferring Survivin siRNA into Bel-7402 cells compared with a non-targeted delivery system, which resulted in more significant gene suppression at both the Survivin mRNA and protein expression levels. Then, the level of caspase-3 activation was significantly elevated, and a remarkable level of tumor cell apoptosis was induced. As a result, the tumor growth in the nude mice Bel-7402 hepatoma model was significantly inhibited. The targeting ability of the RGD-PEG-*g*-PEI-SPION was successfully imaged by MRI scans performed *in vitro* and *in vivo*. Our results strongly indicated that the RGD-PEG-*g*-PEI-SPION can potentially be used as a targeted non-viral vector for altering gene expression in the treatment of hepatocellular carcinoma and for detecting the tumor in vivo as an effective MRI probe.

## Introduction

Hepatocellular carcinoma (HCC) is one of the most common malignant tumors and the third most common cause of cancer-related deaths worldwide [1]. Because of viral infection, diabetes, obesity and excessive alcohol intake, the incidence of HCC is steadily increasing [2]. Conventional surgical management in the early stages of HCC includes local excision, liver transplantation, radiofrequency ablation and ethanol injection. However, more than half of the HCC patients are diagnosed too late to benefit from these curative therapies [3]. Furthermore, the majority of patients with advanced HCC do not have a curative response to palliative treatments, such as radiotherapy and chemotherapy, which can only afford a modest survival benefit for some HCC patients. To attain better treatments for hepatocellular carcinoma, gene therapy has been explored.

The RNA interference (RNAi)-mediated knockdown of protein expression via silencing of the responsible gene at the messenger RNA (mRNA) level provides a promising strategy. Since the discovery of RNAi technology [4], it has been successfully utilized in the treatment of various diseases, especially in the treatment of cancer [5]. Survivin, the smallest member of the inhibitor of apoptosis (IAP) family, is strongly expressed in most human tumors and is not detected in terminally differentiated adult tissues [6]. The Survivin protein is overexpressed in hepatocellular carcinoma and involved in hepatocarcinogenesis. Because Survivin protein overexpression can inhibit apoptosis and accelerate the proliferation of HCC cells [7], [8], we used Survivin small interfering RNA (siRNA) to suppress the expression of the Survivin protein via silencing Survivin mRNA and thus induced the apoptosis of HCC cells. However, the delivery of siRNA into specific tissues and cells is still a barrier.

When injected intravenously, siRNAs are rapidly degraded by extracellular RNases and cleared by renal filtration [9]. Because of their large molecular weight and net negative charge, naked siRNA cannot penetrate the cytomembrane [10]. The development of an efficient delivery system to site-specifically deliver siRNA into tissues or cells is pivotal. Valid siRNA delivery vehicles and small molecules targeted to specific tissues or cells are two critical factors in overcoming this obstacle. Transport vectors have been categorized into two groups: viral and non-viral vectors [11]. In our previous study, we constructed a polymer-based non-viral vector, polyethylene glycol-grafted polyethylenimine functionalized with superparamagnetic iron oxide nanoparticles (PEG-*g*-PEI-SPION) [12]. The polyethyleneimine (PEI) possesses large amounts of functionalized amine groups capable of complexing with nucleic acids. Therefore, PEI can be used to deliver siRNA. To reduce the cytotoxicity induced by the mass of positive charges on PEI and enhance the biocompatibility of the nanoparticles in vivo, the hydrophilic polymer polyethylene glycol (PEG) has been attached to PEI (PEG-*g*-PEI) [13]. Because of low-intensity signals in T_2_-weighted MR imaging, superparamagnetic iron oxide nanoparticles (SPION) have been studied as potential MRI T_2_ probes for imaging tumor cells in vitro and in vivo. Therefore, we introduced the SPION to PEG-*g*-PEI (PEG-*g*-PEI-SPION) and then constructed a MRI-visible non-viral vector that combines the gene therapeutic and MR imaging functions. For the purpose of efficiently targeting siRNA and SPION into specific cells, we chose to conjugate the PEG-*g*-PEI-SPION with a small molecule that specifically binds to the cells of interest. We have successfully modified PEG-*g*-PEI-SPION with various targeting molecules or antibodies that bind to corresponding specific cells [12], [14]. The surface of HCC cells contains an abundance of specific integrin receptors, such as α_ν_β_3_ and α_ν_β_5_, whereas these receptors are rarely expressed in normal hepatocytes [15]. As a specific recognition site on the ligands for α_ν_β_3_, the tripeptide arginine glycine aspartic acid (RGD) can be conjugated to PEG-*g*-PEI-SPION to produce a HCC-targeted therapy.

In the present study, an RGD-modified MRI-visible non-viral vector, polyethylene glycol-grafted polyethylenimine functionalized with superparamagnetic iron oxide nanoparticles (RGD-PEG-*g*-PEI-SPION), was synthesized to specifically deliver Survivin siRNA to HCC cells and sensitively detecting the tumor cells by MRI in vitro and in vivo. We evaluated the physicochemical characteristics of RGD-PEG-*g*-PEI-SPION/siRNA complexes and observed their anti-tumor efficacy after systemic administration of RGD-PEG-*g*-PEI-SPION/siRNA complexes.

## Materials and Methods

### Materials

The synthesis of the PEG-*g*-PEI-SPION was described in detail in our previous publication [12]. RGD was purchased from the Calbiochem Co. (Darmstadt, Germany). The human hepatocellular carcinoma cell line, Bel-7402, was obtained from the Institute of Biochemistry and Cell Biology at the Chinese Academy of Sciences (Shanghai, China). Roswell Park Memorial Institute (RPMI 1640) medium, Penicillin–Streptomycin, Fetal bovine serum (FBS) and Dulbecco's phosphate buffered saline (PBS) were purchased from Gibco BRL (Carlsbad, CA, USA).

3-(4,5-Dimethyl-thiazol-2-yl)-2,5-diphenyltetrazolium bromide (MTT) was obtained from Sigma-Aldrich (St. Louis, MO, USA). The fluorescent staining agent 4′,6-diamidion-2-phenylindole (DAPI) was purchased from Roche (Roche, Germany). Alexa Fluor 555 was purchased from Molecular Probes (Eugene, OR, USA). The fluorescein isothiocyanate (FITC)-labeled siRNA, Survivin siRNA, negative control siRNA (siNC), rabbit anti-human Survivin antibody, rabbit anti-human cleaved caspase-3 antibody and HRP-conjugated goat anti-rabbit antibody were purchased from Cell Signaling Technology (Danvers, CST, USA). Rabbit anti-human CD34 antibody was purchased from Abcam (Cambridge, England). Mouse anti-human α_ν_β_3_ antibody was purchased from Millipore (Billerica, USA).

### Synthesis of RGD-PEG-*g*-PEI-SPION

To construct a targeted siRNA delivery vehicle, RGD (1 mg) was dissolved in 500 µl of 4-(2-hydroxyethyl)-1-piperazineethanesulfonic acid/ethylene diamine tetraacetic acid (HEPES/EDTA) solution (0.5 M, pH 8.0). Hydroxylamine (200 µl) was mixed with HEPES/EDTA (500 µl) and was then added to the RGD solution. After incubation at 37°C for 90 min, three hundred micrograms of heterofunctional PEG terminated with carboxyl and maleimide groups (MAL-PEG-COOH) dissolved in 200 µl of 0.5 M HEPES/EDTA was added to the RGD solution and incubated at 4°C overnight. Ten micrograms of both *N*-hydroxysuccinimide (NHS) and 1-ethyl-3-(3-dimethylaminopropyl) carbodiimide (EDC) was added to the RGD-functionalized PEG (RGD-PEG-COOH) solution and incubated at 4°C for 10 min. PEG-*g*-PEI-SPION (100 µl, 1 µg/µl) was subsequently added, and the solution was incubated at 4°C overnight. The final solution was washed three times with PBS (that contained 0.5 M EDTA) in an Amicon cell (MWCO = 10 kDa). The final product, RGD-PEG-*g*-PEI-SPION, was then recovered. (Text S1)

### Agarose Gel Electrophoresis Studies

The degree of binding between siRNA and the non-viral vector was determined with agarose gel electrophoresis. Both PEG-*g*-PEI-SPION/siRNA and RGD-PEG-*g*-PEI-SPION/siRNA complexes were prepared at various N/P ratios (nitrogen of non-viral vector/phosphate of siRNA) that ranged from 1 to 3. The amount of Survivin siRNA for each sample was 50 pmol. After the complexes were incubated for 30 min at room temperature, they were loaded onto the 1% agarose gels with ethidium bromide (0.1 mg/ml) and run with Tris-acetate (TAE) buffer at 100 V for 30 min. The retardation of siRNA mobility was visualized using an ultraviolet (UV) imaging system (DNR Bio-Imaging Systems, Israel).

### Zeta Potential and Particle Size Distribution Measurements

Both PEG-*g*-PEI-SPION/siRNA and RGD-PEG-*g*-PEI-SPION/siRNA complexes were prepared at various N/P ratios that ranged from 3 to 80. The amount of Survivin siRNA for each sample was 100 pmol. After each sample was incubated for 30 min at room temperature, the surface charge and particle size of each sample were analyzed with a Zeta-Plus instrument (Brookhaven Instruments Corporation, USA) at 25°C. The data represent the averages±standard deviations. All tests were performed in triplicate.

### Cell Cultures

Human hepatocellular carcinoma Bel-7402 cells were grown and maintained in RPMI 1640 medium, supplemented with 10% FBS, 100 U/mL penicillin, and 100 U/mL streptomycin, and the cells were maintained at 37°C under a humidified atmosphere of 5% CO_2_.

### Immunocytochemistry Assay

Bel-7402 cells were cultured on the small square glass coverslips in a 6-well plate. 24 h later, cells were washed with phosphate-buffered saline (PBS) and fixed with 95% methanol for 20 min. After washing with PBS, cells were incubated in 0.3% hydrogen peroxide for 30 min and then washed twice with PBS. After being blocked with 10% normal goat whole serum for 60 min, cells were incubated with mouse anti-human α_ν_β_3_ antibody (1:500 dilution) overnight at 4°C. Then, the cells were washed with PBS, further incubated for 30 min with HRP-conjugated goat anti-mouse antibody, and then stained with 3,3-diaminobenzidine (DAB). The cells as negative control received only PBS without first antibody.

### Cell Transfection Analysis

Bel-7402 cells were seeded into a 6-well plate at a density of 1×10^6^ cells per well 24 h prior to siRNA transfection. The FITC-labeled siRNA was used to evaluate the transfection efficiency of various complexes. The concentration of siRNA applied per well in the cell culture was set to 100 nM. The RGD-PEG-*g*-PEI/siRNA and PEG-*g*-PEI/siRNA complexes were prepared at various N/P ratios that ranged from 5 to 35. After the various complexes were mixed for 30 min at room temperature, they were added to each well and further incubated with the cells for 4 h at 37°C in a CO_2_ incubator. All processes were conducted in the dark. After the cells were incubated with the vectors, they were detached and resuspended in 300 µl PBS. Cells cultured as usual without transfection were used as a control for background calibration. The transfection efficiency was evaluated with a FACScan flow cytometer (Becton Dickinson, Franklin Lakes, NJ, USA). The WinMDI software package (version 2.9, Joseph Trotter, Scripps Research Institute, La Jolla, CA, USA) was used to analyze the data. The cells were observed under the fluorescence microscope (Nikon, Tokyo, Japan), and images were concurrently recorded. With respect to the free-ligand competitive inhibition assay, cells were pre-treated with free RGD (10 mM) for 30 min before RGD-PEG-*g*-PEI-SPION/siRNA was added to the culture medium.

### Cell Uptake Analysis

The uptake of complexes by the cells was evaluated by confocal laser scanning microscopy (CLSM) experiments using a Zeiss LSM 510 META microscope (Carl Zeiss, Göttingen, Germany). The RGD-PEG-*g*-PEI-SPION and PEG-*g*-PEI-SPION were dyed with Alexa Fluor 555, as reported previously.[14] The Bel-7402 cells were seeded at a density of 1×10^3^ cells in a confocal dish. The FITC-labeled siRNA was combined with the corresponding amount of RGD-PEG-*g*-PEI-SPION or PEG-*g*-PEI-SPION at a N/P ratio of 10. The siRNA concentration per well was set to 100 nM. After a 30 min incubation at room temperature, the prepared complexes were added to each confocal dish. The DNA-staining agent DAPI (1 mg/ml) was added 4 h later, the confocal dish was further incubated for 15 min, and the CLSM observations were subsequently performed. The free-ligand competitive inhibition assay was also performed.

### Mtt Assay

The evaluation of cytotoxicity was performed with the MTT assay. Bel-7402 cells were seeded at a density of 5×10^3^ cells per well in 96-well plates. Each well contained 100 µl of RPMI-1640 medium supplemented with 10% FBS, and the Bel-7402 cells were cultured for 24 h at 37°C. The cells were then incubated for 48 h with the following complexes: RGD-PEG-*g*-PEI-SPION complexed with Survivin siRNA (RGD-PEG-*g*-PEI-SPION/siRNA); RGD-PEG-*g*-PEI-SPION complexed with negative control siRNA (RGD-PEG-*g*-PEI-SPION/siNC); PEG-*g*-PEI-SPION complexed with Survivin siRNA (PEG-*g*-PEI-SPION/siRNA); and PEG-*g*-PEI-SPION complexed with negative control siRNA (PEG-*g*-PEI-SPION/siNC). All the complexes were prepared at various N/P ratios ranging from 3 to 35. The siRNA concentration in each well was set to 100 nM. After incubation with the complexes, the medium was replaced with 100 µl of complete medium, and 10 µl of MTT solution (5 mg/ml) in PBS was added to each well. After the cells were further incubated for 4 h, the MTT-containing medium was removed and 110 µl of dimethylsulfoxide (DMSO) was added to each well. After the cells were gently agitated for 5 min, the absorbance of each well was recorded with an Infinite F200 Multimode plate reader (Tecan, Crailsheim, Germany) at 490 nM. All experiments were conducted in triplicate. The data represent the averages ±standard deviations.

### Molecular Biology Assays *in vitro*

Bel-7402 cells were grown to 90% confluency in 6-well plates and incubated with RGD-PEG-*g*-PEI-SPION/siRNA, PEG-*g*-PEI-SPION/siRNA, RGD-PEG-*g*-PEI-SPION/siNC or PEG-*g*-PEI-SPION/siNC at a N/P ratio of 10. The applied Survivin siRNA and negative control siRNA concentrations per well were 100 nM. The level of *Survivin* gene mRNA in the transfected Bel-7402 cells was evaluated with real-time polymerase chain reaction (RT-PCR). After the cells were incubated for 48 h, the total RNA was isolated from the transfected cells using the TRIzol reagent (Invitrogen, Carlsbad, CA, USA). RT-PCR was performed using the PrimeScript RT-PCR Kit according to the manufacturer's protocol. The specific oligonucleotide primers targeting the Survivin sequence were 5′-CAGACTTGGCCCAGTGTTTC-3′ for the forward primer and 5′-CACTTTCTCCGCAGTTTCCTC-3′ for the reverse primer. The mRNA level of â-actin gene was measured in each sample as an internal normalization standard. The forward primer was 5′- CCAACCGCGAGAAGATGA-3′, and the reverse primer was 5′- CCAGAGGCGTACAGGGATAG-3′.The PCR was run on a StepOne Plus Real-time PCR System (ABI, USA). All experiments were performed in triplicate. The PCR reaction involved heating at 95°C for 5 minutes, 30 cycles at 95°C for 15 seconds, 60°C for 30 seconds, and 72°C for 35 seconds.

The expression of Survivin protein and cleaved caspase-3 protein were evaluated with western blotting. After the cells were incubated for 48 h, total protein was extracted using the ProteoJET™ Mammalian Cell Lysis Reagent (Fermentas, Canada) with phenylmethanesulfonyl fluoride (PMSF). The protein content was determined using the bicinchoninic acid protein assay kit (Invitrogen, Carlsbad, CA, USA). Ten micrograms of protein was separated on a 10% sodium dodecyl sulfate (SDS) polyacrylamide gel and transferred to polyvinylidene difluoride (PVDF) membranes. The membranes were blocked with 5% nonfat milk in Tris-buffered saline Tween-20 (TBST) for 1 h at room temperature and were subsequently incubated with rabbit anti-human Survivin antibody (1:1000 dilution in PBS/Tween; Cell Signaling Technology, Danvers, USA) and rabbit anti-human cleaved caspase-3 antibody (1:1000 dilution in PBS/Tween; Cell Signaling Technology, Danvers, USA) overnight at 4°C. After the membranes were washed twice with TBST, they were incubated with HRP-conjugated goat anti-rabbit antibody diluted 1:5000 in TBST buffer for 1 h at room temperature. An enhanced chemiluminescence detection reagent (Millipore, USA) was then used, and the protein bands were visualized after exposure to X-ray film. Cells cultured as usual without transfection were used as a control.

### Cell Apoptosis Assays *in vitro*

Bel-7402 cells were seeded at a density of 7×10^3^ cells per well in a 24-well plate and incubated overnight at 37°C followed by the addition of the complexes that were prepared as described in the previous section. After the cells were incubated for 48 h, the terminal deoxynucleotidyltransferase-mediated UTP end-labeling (TUNEL) assay was performed according to the manufacturer's protocol for the In Situ Cell Death Detection Kit with Tetramethylrhodamine (TMR) red (Roche, Mannheim, Germany). Briefly, the cells were fixed with freshly prepared 4% paraformaldehyde in PBS (pH 7.4) for 1 h and then washed twice with PBS (pH 7.4). The permeabilization solution, 0.1% Triton X-100 in 0.1% sodium citrate, was added to each well, and the cells were incubated for 2 min on ice. The cells were washed twice with PBS (pH 7.4). Then, 50 µl of the TUNEL reaction mixture was added to each well, and the cells were incubated for 1 h at 37°C in a humidified atmosphere in the dark. The cells were washed three times with PBS (pH 7.4). After the cells were incubated for an additional 15 min with the DNA-staining agent DAPI (1 mg/ml), they were analyzed using an inverted fluorescence microscope (Nikon, Tokyo, Japan). Cells cultured as usual without transfection were used as a control.

### Mri Analysis of The *in vitro* Targeting of Cells

Bel-7402 cells, seeded at a density of 2×10^6^ cells per well in 6-well plates, were incubated for 1 h in the presence of RGD-PEG-*g*-PEI-SPION or PEG-*g*-PEI-SPION at Fe concentrations of 10, 15, 30, 50, and 70 µg/ml in complete RPMI-1640 medium. After the Bel-7402 cells were washed three times with PBS, they were detached and resuspended in a 4% gelatin solution. The cells were scanned under a 1.5 T MR scanner (GE Healthcare UK Limited, Buckinghamshire, UK), and a wrist coil with an inner diameter of 3 inches was used. Spin echo T_2_-weighted images (T_2_WI) were acquired using the following parameters: TR/TE, 2000/103 ms; FOV, 200 mm; matrix, 320×224; slice thickness, 2.0 mm. The signal intensities (SI) of the cells were assessed using a circular 30 mm^2^ region of interest. The SI of the treated cells were normalized by comparison with the SI of the control cells. The initial T_2_-mapping images were acquired using the following parameters: TR, 2500 ms; TE, 14 ms, 28 ms, 42 ms, and 56 ms; FOV, 200 mm; matrix, 224×192; slice thickness, 2.0 mm. The T_2_-mapping images and the T_2_ values of the treated cells were calculated using the software tools provided by the manufacturer. Cells cultured as usual without transfection were used as a control.

### The Nude Mice Bel-7402 Hepatoma Model

Four-week-old female BALB/c nude mice (15–20 g) were purchased from the animal center of Sun Yat-sen University (Guangzhou, China). All experiments were performed in accordance with the National Institutes of Health Guide for Care and Use of Laboratory Animals and were approved by the Bioethics Committee of Sun Yat-sen University. All nude mice were bred by the professionals in Sun Yat-sen University. The nude mice were injected subcutaneously in the right thigh with 100 µl of a single-cell suspension that contained 4×10^6^ Bel-7402 cells. Five days later, the tumor volumes were ∼18–20 mm^3^. The mice were randomly divided into five groups (10 mice per group) for the tumor growth inhibition studies. The tumor size was measured with a digital vernier caliper across its longest (a) and shortest (b) diameters, and its volume was calculated using the formula V = 0.5ab^2^.

### Studies on The Inhibition of Tumor Growth

All of the nude mice bearing a Bel-7402 subcutaneous tumor were injected with complexes via the tail vein every other day according to the following groupings: Group 1: Control; Group 2: RGD-PEG-*g*-PEI-SPION/siRNA; Group 3: PEG-*g*-PEI-SPION/siRNA; Group 4: RGD-PEG-*g*-PEI-SPION/siNC; Group 5: PEG-*g*-PEI-SPION/siNC. The injection doses of the Survivin siRNA and negative control siRNA were 1.6 mg siRNA/kg body weight. All the complexes were formed at a N/P ratio of 10. In all cases, the tumors were measured once every 4 days for up to 25 days after the first injection. The tumor-bearing nude mice injected with PBS were used as controls.

### Histology and Immunohistochemistry

The mice were sacrificed, and the tumors were removed. After being fixed in 4% paraformaldehyde for 24 h, the tumors were embedded in paraffin. After deparaffinization, tumor tissue sections were stained with hematoxylin/eosin (H&E). Immunohistochemical analysis of the tumor tissue was performed. In brief, tumor tissue sections were subjected to deparaffinization, rehydration, and antigen retrieval, in sequence, and then incubated with 3% hydrogen peroxide for 30 min to inactivate the endogenous peroxidases. After being blocked with protein blocking serum, the tumor tissue sections were incubated with rabbit anti-human Survivin antibody (1:400 dilution), rabbit anti-human cleaved caspase-3 antibody (1:800 dilution) and rabbit anti-human CD34 antibody (1:200 dilution) overnight at 4°C. Then, the tumor tissue sections were washed with PBS, further incubated for 1 h with HRP-conjugated goat anti-rabbit antibody, and then stained with 3,3-diaminobenzidine (DAB). Finally, the tumor tissue sections were washed with PBS and stained with hematoxylin.

### Cell Apoptosis Assays *in vivo*

The evaluation of cell apoptosis in vivo was performed on the excised tumor tissues using an In Situ Cell Death Detection Kit with POD (Roche, Mannheim, Germany) according to the manufacturer's protocol. In brief, the tumor tissue sections were treated with deparaffinization and rehydration (in sequence) and were then incubated with proteinase K (20 µg/ml in 10 mm Tris/HCl, pH 8.0) for 20 min at room temperature. After the tumor tissue sections were washed twice with PBS, they were incubated for 10 min with the permeabilization solution, 0.1% Triton X-100 in 0.1% sodium citrate. Subsequently, the tumor tissue sections were incubated with the TUNEL reaction mixture for 2 h at 37°C in a humidified atmosphere in the dark. After the tumor tissue sections were washed twice with PBS, they were treated with 3% hydrogen peroxide for 30 min to inactivate the endogenous peroxidases. Next, the tumor tissue sections were incubated with a Converter-POD solution for 30 min at 37°C and then further stained with 3,3-diaminobenzidine (DAB) for 10 min at room temperature. Finally, the tumor tissue sections were washed with PBS and stained with hematoxylin.

### MRI Analysis of the *in vivo* Targeting of Cells

The preparation of nude mice bearing Bel-7402 subcutaneous tumors was performed as described in the previous section. Ten days after tumor initiation, the tumor size attained a volume of ∼55–60 mm^3^, and the mice were randomly divided into two groups (5 mice per group) for MR imaging. After anesthetization with 10% chloral hydrate (5 µl/g), the mice bearing the Bel-7402 tumor were scanned using a 1.5 T MR scanner (GE Healthcare UK, Buckinghamshire, UK) and a 5-cm linearly polarized birdcage radio frequency mouse coil was used. The mice were subsequently injected with RGD-PEG-*g*-PEI-SPION or PEG-*g*-PEI-SPION (10 mg Fe/kg body weight) via tail vein according to grouping. Three hours later, the same MR imaging was repeated. T_2_WI was acquired using the following parameters: TR/TE, 2000/88 ms; FOV, 70 mm; matrix, 320×192; slice thickness, 1.0 mm. The signal intensities (SI) of the tumors were assessed using a circular 30 mm^2^ region of interest. The SI of the tumors after injection were normalized to those of the tumors before injection. The initial T_2_-mapping images were acquired using the following parameters: TR, 2500 ms; TE, 14 ms, 28 ms, 42 ms, and 56 ms; FOV, 70 mm; matrix, 224×192; slice thickness, 1.0 mm. T_2_-mapping images and T_2_ values of tumors were calculated with the software tools provided by the manufacturer.

### Prussian Blue Staining

The tumor tissue sections were prepared as described in the previous section. The sections were rinsed in distilled water, incubated for 30 min in a 1:1 solution of 2% aqueous potassium ferrocyanide and 2% hydrochloric acid, rinsed and then counterstained with 1% neutral red.

### Statistical Analysis

The results are expressed as the means ± the standard deviations (SDs). Statistical analysis of the data was performed with the one-factor analysis of variance (SPSS software, version 13.0, SPSS). A *P*-value<0.05 was considered statistically significant. All statistical tests were two-sided.

## Results

### Agarose Gel Electrophoresis

Agarose gel electrophoresis was conducted to confirm the siRNA condensation ability of the non-viral vectors, PEG-*g*-PEI-SPION and RGD-PEG-*g*-PEI-SPION. Naked siRNA was used as a control. The negatively charged siRNA can move in the electric field. The positive charges of the non-viral vectors neutralize the negative charges of the siRNA, thus retarding the siRNA mobility. As shown in Figure S1, the siRNA bands decreased with increasing N/P ratios. When the N/P ratio of RGD-PEG-*g*-PEI-SPION/siRNA and PEG-*g*-PEI-SPION/siRNA reached 2.5, free siRNA could not be detected, which indicated that the negatively charged siRNA could be neutralized entirely at a N/P ratio of 2.5.

### Zeta Potential and Particle Size

The zeta potentials and particle sizes of RGD-PEG-*g*-PEI-SPION/siRNA and PEG-*g*-PEI-SPION/siRNA were studied to deliver siRNA with high efficiency. As shown in Figure S2A, the zeta potential and particle size of pure RGD-PEG-*g*-PEI-SPION were +27.0±1.1 mV and 63.0±6.1 nm, respectively. After RGD-PEG-*g*-PEI-SPION was complexed with siRNA at a N/P ratio of 3, the zeta potential of the RGD-PEG-*g*-PEI-SPION/siRNA decreased abruptly to +3.0±1.7 mV, and the particle size increased abruptly to 206.0±1.9 nm. With increasing N/P ratios, the zeta potential of the RGD-PEG-*g*-PEI-SPION/siRNA increased gradually and reached +12.5±1.3 mV at a N/P ratio of 10. In addition, the particle size decreased gradually and attained a constant size of 85.1±5.7 nm at a N/P ratio of 10. With respect to the PEG-*g*-PEI-SPION/siRNA, the transformation rules of zeta potential and particle size were in accord with what was observed with RGD-PEG-*g*-PEI-SPION/siRNA. As shown in Figure S2B, the zeta potential and particle size of pure PEG-*g*-PEI-SPION were +31.0±2.1 mV and 60.2±7.1 nm, respectively. At a N/P ratio of 3, the zeta potential and particle size of PEG-*g*-PEI-SPION/siRNA were +6.1±1.6 mV and 198.8±2.7 nm, respectively. At a N/P ratio of 10, the zeta potential of PEG-*g*-PEI-SPION/siRNA was +14.0±1.2 mV, and the particles attained a constant size of 83.1±6.7 nm.

### Receptor Expression Assay

The Immunocytochemistry assay was performed to study the expression of α_ν_β_3_ in Bel-7402 cells. As shown in Figure S3, the brown stains represented the α_ν_β_3_ expressed on Bel-7402 cells. The results showed that cell membranes of the Bel-7402 cells possessed an enrichment of α_ν_β_3_ receptors.

### *In vitro* Transfection Assay of Bel-7402 Cells

**Figure 1:**
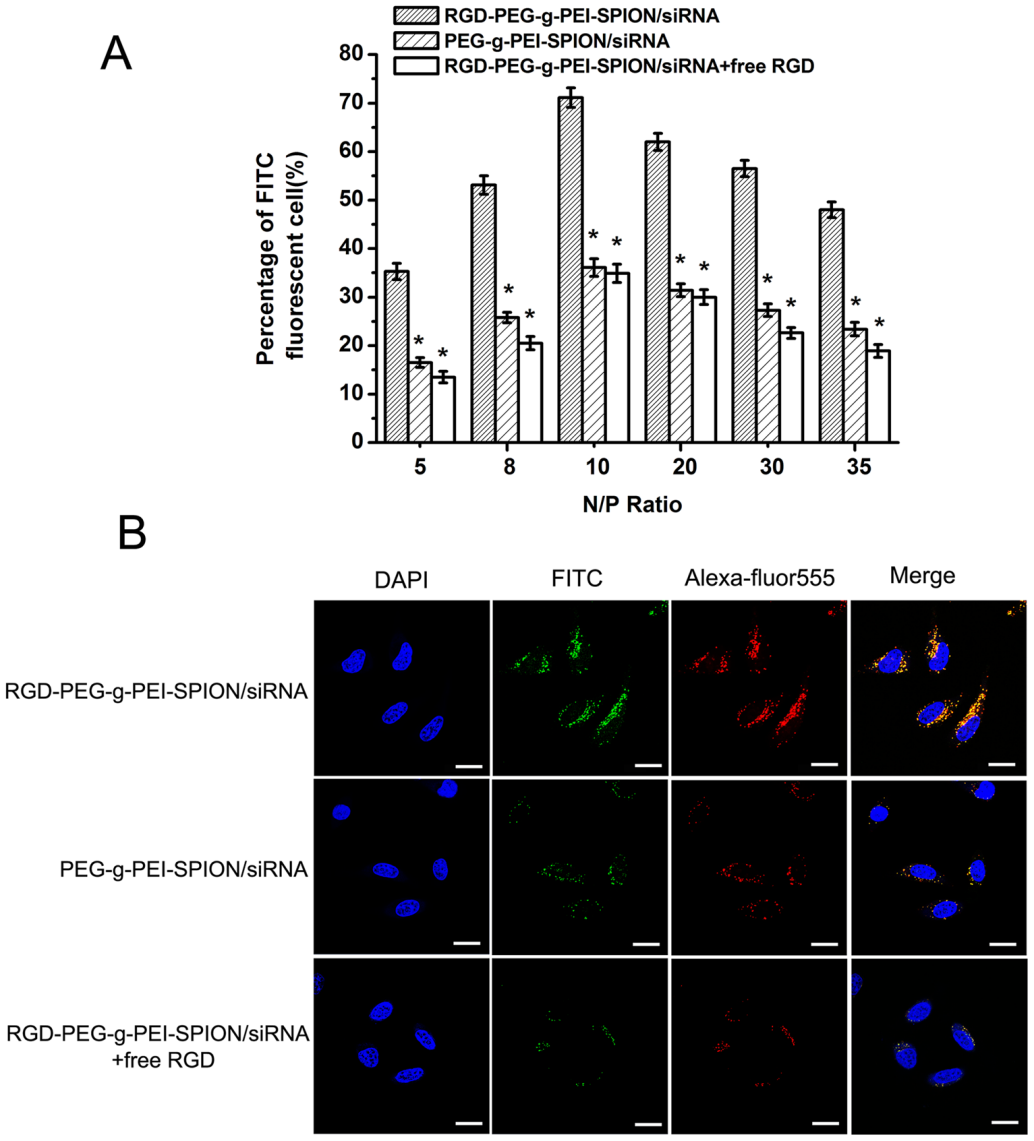
*In vitro* cell transfection efficiency analysis and cell uptake analysis. (A) The percentage of FITC-positive cells incubated with RGD-PEG-*g*-PEI-SPION/siRNA, PEG-*g*-PEI-SPION/siRNA or RGD-PEG-*g*-PEI-SPION/siRNA in the presence of free RGD at various N/P ratios. (means±SD, n = 3; *p<0.05, compared with RGD-PEG-*g*-PEI-SPION/siRNA at the same N/P ratio). (B) The laser confocal microscope images of Bel-7402 cells transfected with RGD-PEG-*g*-PEI-SPION/siRNA, PEG-*g*-PEI-SPION/siRNA or RGD-PEG-*g*-PEI-SPION/siRNA in the presence of free RGD at a N/P ratio of 10. (×630; scale bar:10 µm).

The ability of various complexes to deliver siRNA into Bel-7402 cells was evaluated by flow cytometry and fluorescence microscopy. As shown in Figure 1A, the percentage of FITC-positive cells was different at various N/P ratios. However, at the same N/P ratio, the percentage of FITC-positive cells incubated with RGD-PEG-*g*-PEI-SPION/siRNA was always significantly higher than that of cells incubated with PEG-*g*-PEI-SPION/siRNA or RGD-PEG-*g*-PEI-SPION/siRNA in the presence of free RGD (*p*<0.05). The percentage of FITC-positive cells incubated with RGD-PEG-*g*-PEI-SPION/siRNA, PEG-*g*-PEI-SPION/siRNA and RGD-PEG-*g*-PEI-SPION/siRNA with free RGD all attained their highest values at a N/P ratio of 10, (Figure S4A), which were 71.1%±2.0%, 36.1%±1.8% and 34.9%±1.9%, respectively. The fluorescence images of Bel-7402 cells incubated with the various complexes are shown in Figure S4B. The green fluorescence intensity of the cells incubated with RGD-PEG-*g*-PEI-SPION/siRNA was significantly stronger than that of the cells incubated with the other complexes at a N/P ratio of 10, which indicates that more complexes were internalized into the cells.

### *In vitro* Assay For Uptake Into Bel-7402 Cells

The cellular uptake ability of various complexes was evaluated with laser confocal microscopy. The FITC-labeled siRNA (green fluorescence) was used to visualize the cellular uptake of siRNA. The Alexa Fluor 555 (red fluorescence) was used to label the non-viral vectors to visualize the cellular uptake of RGD-PEG-*g*-PEI-SPION and PEG-*g*-PEI-SPION. The nuclei were stained with DAPI (blue fluorescence). As shown in Figure 1B, the cells incubated with RGD-PEG-*g*-PEI-SPION/siRNA showed significantly stronger RGD-PEG-*g*-PEI-SPION (red) and siRNA (green) fluorescence in comparison with the cells incubated with PEG-*g*-PEI-SPION/siRNA or RGD-PEG-*g*-PEI-SPION/siRNA in the presence of free RGD at a N/P ratio of 10. The overlapping of the red and green fluorescence of cells incubated with RGD-PEG-*g*-PEI-SPION/siRNA generated yellow stains in the merged images. These results indicate that the RGD-modified non-viral vector, RGD-PEG-*g*-PEI-SPION, can deliver more siRNA into Bel-7402 cells compared to the PEG-g-PEI-SPION. Furthermore, the confocal images revealed the intracellular distributions of non-viral vectors and siRNA fluorescence in the cytoplasm and periphery of the nuclei.

### Bel-7402 Cell Viability Assay

**Figure 2:**
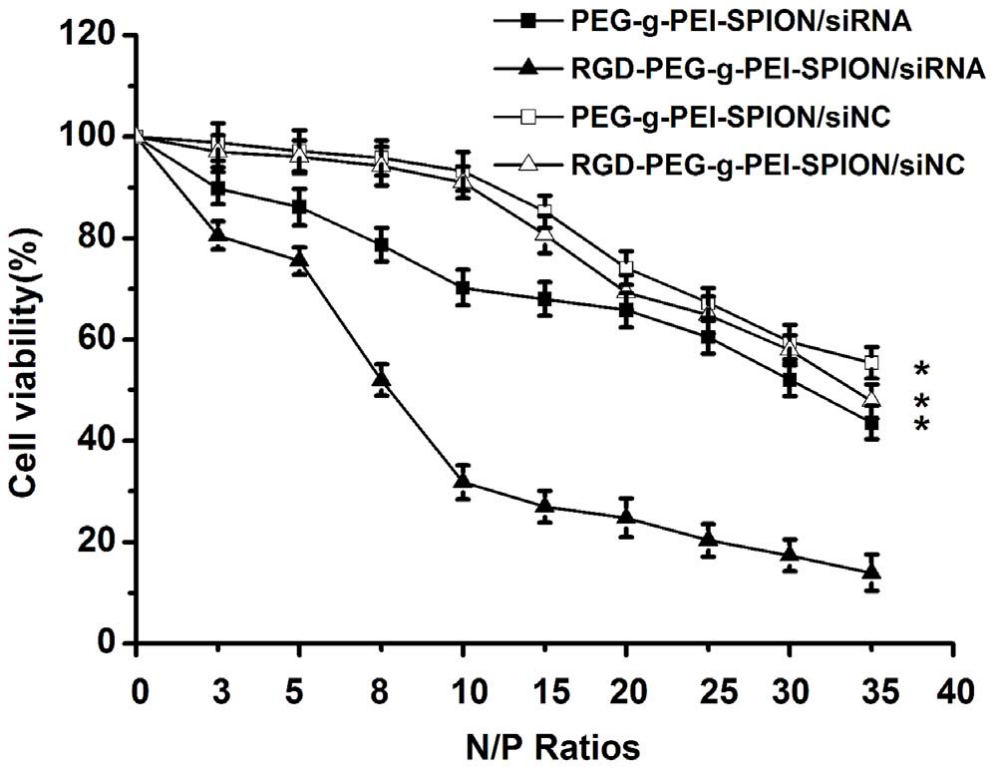
The cytotoxicities of various complexes in Bel-7402 cells. The viability of Bel-7402 cells incubated with RGD-PEG-*g*-PEI-SPION/siRNA, PEG-*g*-PEI-SPION/siRNA, RGD-PEG-*g*-PEI-SPION/siNC or PEG-*g*-PEI-SPION/siNC at various N/P ratios. (means±SD; n = 3; *p<0.05, compared with RGD-PEG-*g*-PEI-SPION/siRNA).

The influence of various complexes on the viability of Bel-7402 cells was analyzed using an MTT assay. Cells cultured as usual without transfection were used as a control, and the cell viability of the control was considered to be 100%. As shown in Figure 2, the cytotoxicity of the various complexes all gradually increased with increasing N/P ratios. At a N/P ratio of 10, the viability of cells incubated with the RGD-PEG-*g*-PEI-SPION/siRNA, PEG-*g*-PEI-SPION/siRNA, RGD-PEG-*g*-PEI-SPION/siNC or PEG-*g*-PEI-SPION/siNC was 31.8%±3.4%, 70.2%±3.5%, 91.0%±3.1% and 93.2%±3.8%, respectively. Furthermore, the RGD-PEG-*g*-PEI-SPION/siRNA exhibited higher cytotoxicity at all N/P ratios than did the other complexes (*p*<0.05), which indicates that the binding of RGD enhanced the delivery of Survivin siRNA.

### *In vitro Survivin* Gene Suppression Assay

**Figure 3:**
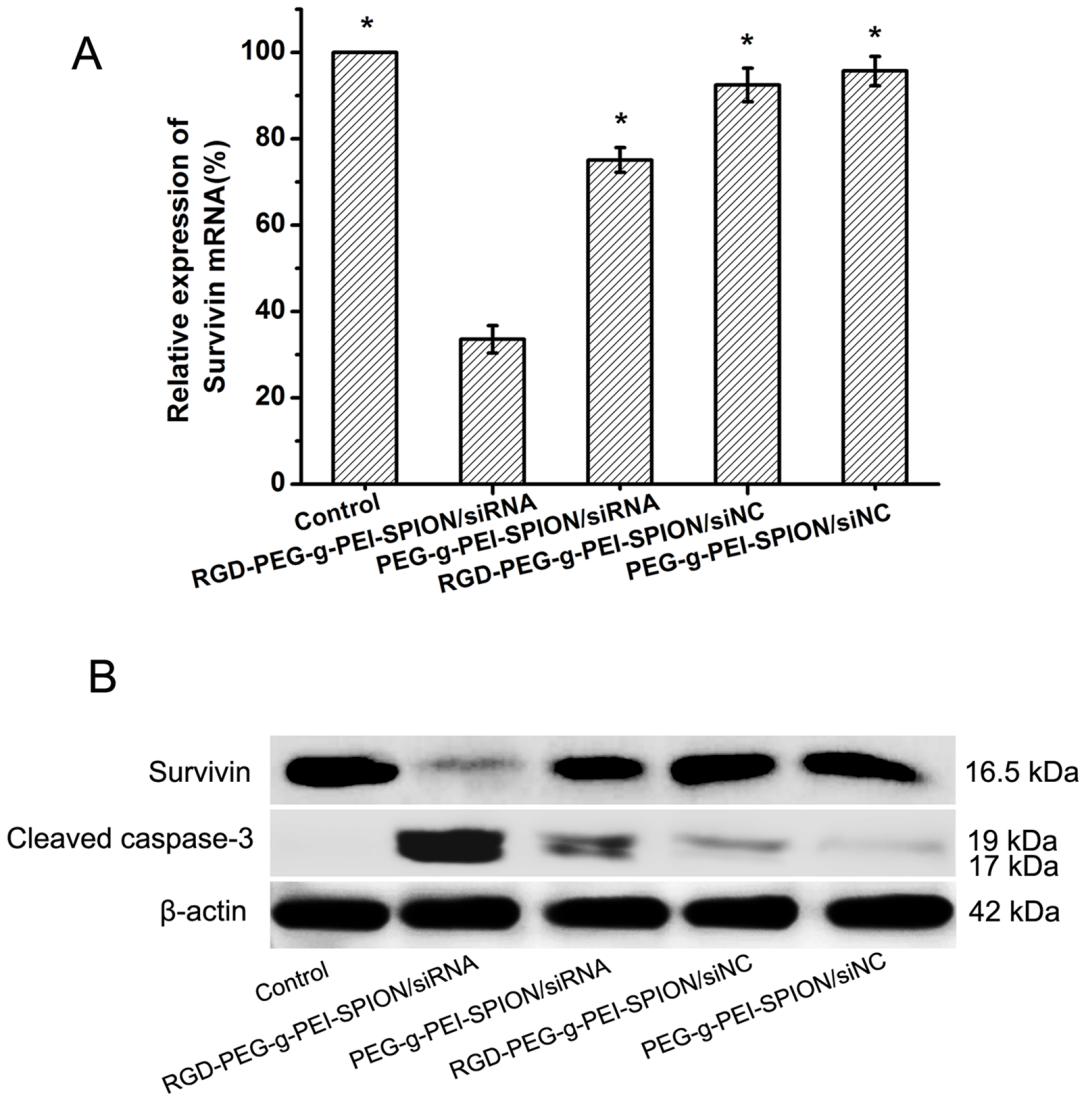
Efficacy of different treatments in suppressing Survivin gene expression in Bel-7402 cells. (A) Efficacy of RGD-PEG-*g*-PEI-SPION/siRNA, PEG-*g*-PEI-SPION/siRNA, RGD-PEG-*g*-PEI-SPION/siNC or PEG-*g*-PEI-SPION/siNC in suppressing *Survivin* gene expression in Bel-7402 cells at a N/P ratio of 10 as quantified by RT-PCR analysis (means±SD; n = 3; *p<0.05, compared with RGD-PEG-*g*-PEI-SPION/siRNA; Control: the cells without transfection). (B) The expression of Survivin and cleaved caspase-3 protein in Bel-7402 cells incubated with various complexes evaluated by western blot analysis.

The ability of various complexes (RGD-PEG-*g*-PEI-SPION/siRNA, PEG-*g*-PEI-SPION/siRNA, RGD-PEG-*g*-PEI-SPION/siNC and PEG-*g*-PEI-SPION/siNC) to reduce the levels of Survivin mRNA and protein expression in Bel-7402 cells was analyzed using RT-PCR and western blot analysis, respectively. As shown in Figure 3A, at a N/P ratio of 10, the relative percentages for Survivin mRNA expression of the cells incubated with RGD-PEG-*g*-PEI-SPION/siRNA, PEG-*g*-PEI-SPION/siRNA, RGD-PEG-*g*-PEI-SPION/siNC or PEG-*g*-PEI-SPION/siNC were 33.6%±3.2%, 75.1%±2.9%, 92.5%±3.9% and 95.7%±3.4%, respectively. The RGD-PEG-*g*-PEI-SPION/siRNA exhibited the greatest effect on *Survivin* gene suppression in Bel-7402 cells (*p*<0.05) in comparison with the other complexes, which indicates that Survivin siRNA could effectively suppress the expression of *Survivin* at the mRNA level, whereas the negative control siRNA had no obvious inhibitory effect. In addition, these results further verify that the conjugation of RGD to the vectors could enhance the ability of the complexes to deliver Survivin siRNA into Bel-7402 cells.

The *Survivin* gene suppression effect was further confirmed by western blot analysis. As shown in Figure 3B, compared with the Survivin protein band expressed by the cells incubated with the other complexes, the Survivin protein band expressed by the cells incubated with RGD-PEG-*g*-PEI-SPION/siRNA were weaker, which is in accordance with the results from RT-PCR. In addition, the cells treated the same way expressed the strongest cleaved caspase-3 protein band, which is a good indicator of cell apoptosis; this result indicates that the down-regulation of *Survivin* gene expression could induce cell apoptosis.

### The Ability of Survivin siRNA to Induce cell Apoptosis *in vitro*

**Figure 4:**
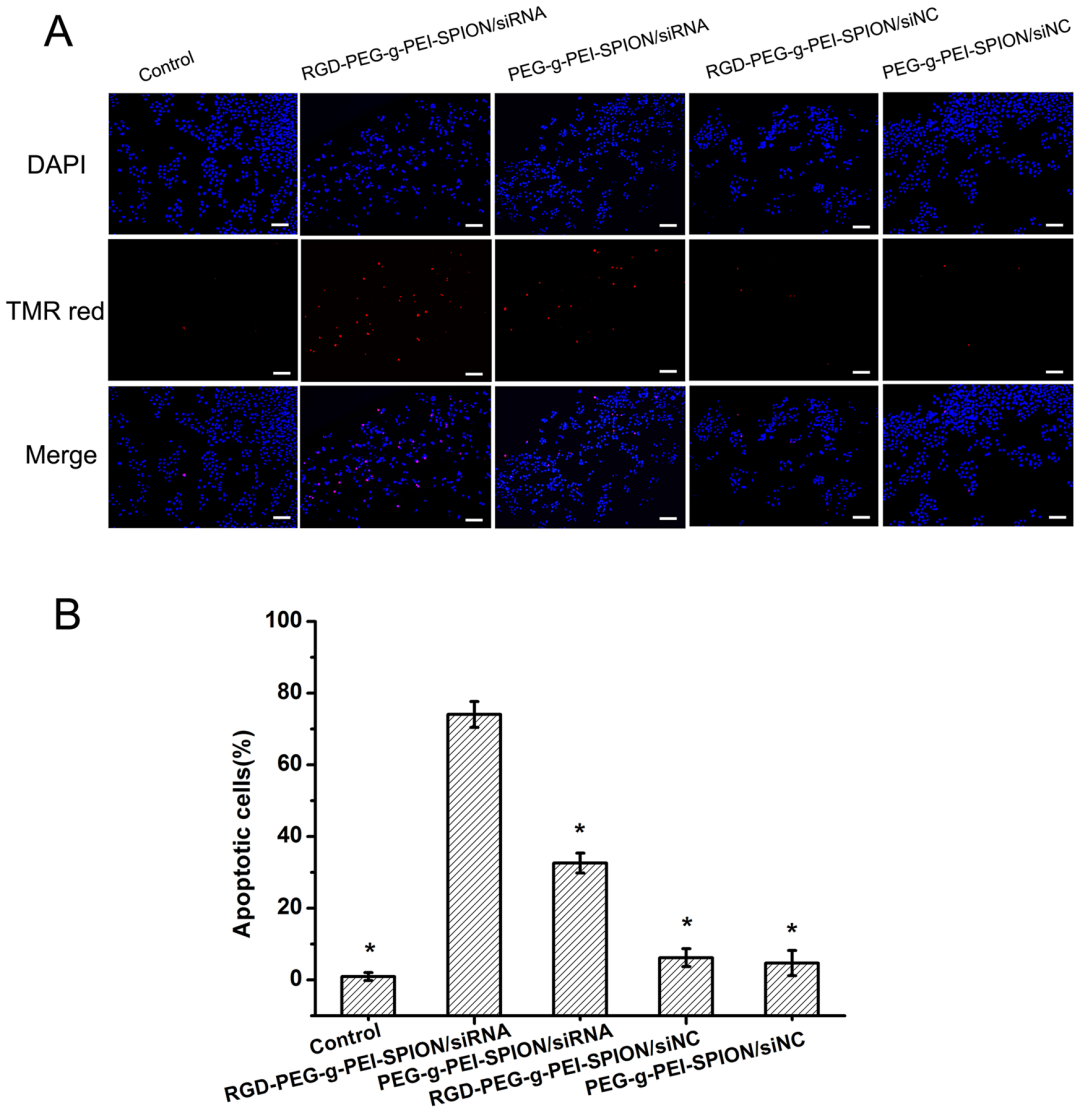
The apoptotic response to different treatments in Bel-7402 cells. (A) Images show Bel-7402 cells incubated with: RGD-PEG-*g*-PEI-SPION/siRNA, PEG-*g*-PEI-SPION/siRNA, RGD-PEG-*g*-PEI-SPION/siNC or PEG-*g*-PEI-SPION/siNC (×200; scale bar:100 µm) (B) Percentage of apoptotic cells induced by various complexes at N/P ratio of 10 as quantified by TUNEL analysis (means±SD; n = 3; *p<0.05, compared with RGD-PEG-*g*-PEI-SPION/siRNA; Control: the cells without transfection).

The TUNEL assay was performed to verify the therapeutic effect of Survivin siRNA. As shown in Figure 4A, the cells with nuclei that contained amaranth fluorescence in the merged images represented apoptotic cells, i.e., TUNEL-positive cells. The percentage of apoptotic cells was obtained via counting the number of TUNEL-positive cells. The Bel-7402 cells incubated with RGD-PEG-*g*-PEI- SPION/siRNA showed the highest level of cell apoptosis (*p*<0.05) compared with the cells incubated with the other complexes; the percentage of apoptotic cells was 74.1%±3.6%. However, the Bel-7402 cells incubated with PEG-*g*-PEI- SPION/siRNA, RGD-PEG-*g*-PEI-SPION/siNC and PEG-*g*-PEI-SPION/siNC resulted in percentages of cell apoptosis of 32.6%±2.8%, 6.2%±2.5% and 4.7%±3.5%, respectively (Figure 4B). The TUNEL assay data, which are consistent with the MTT, RT-PCR and western blot results, again illustrate that RGD-PEG-*g*-PEI-SPION delivers Survivin siRNA into Bel-7402 cells with high efficiency and induces cell apoptosis.

### *In vitro* MR Imaging

**Figure 5:**
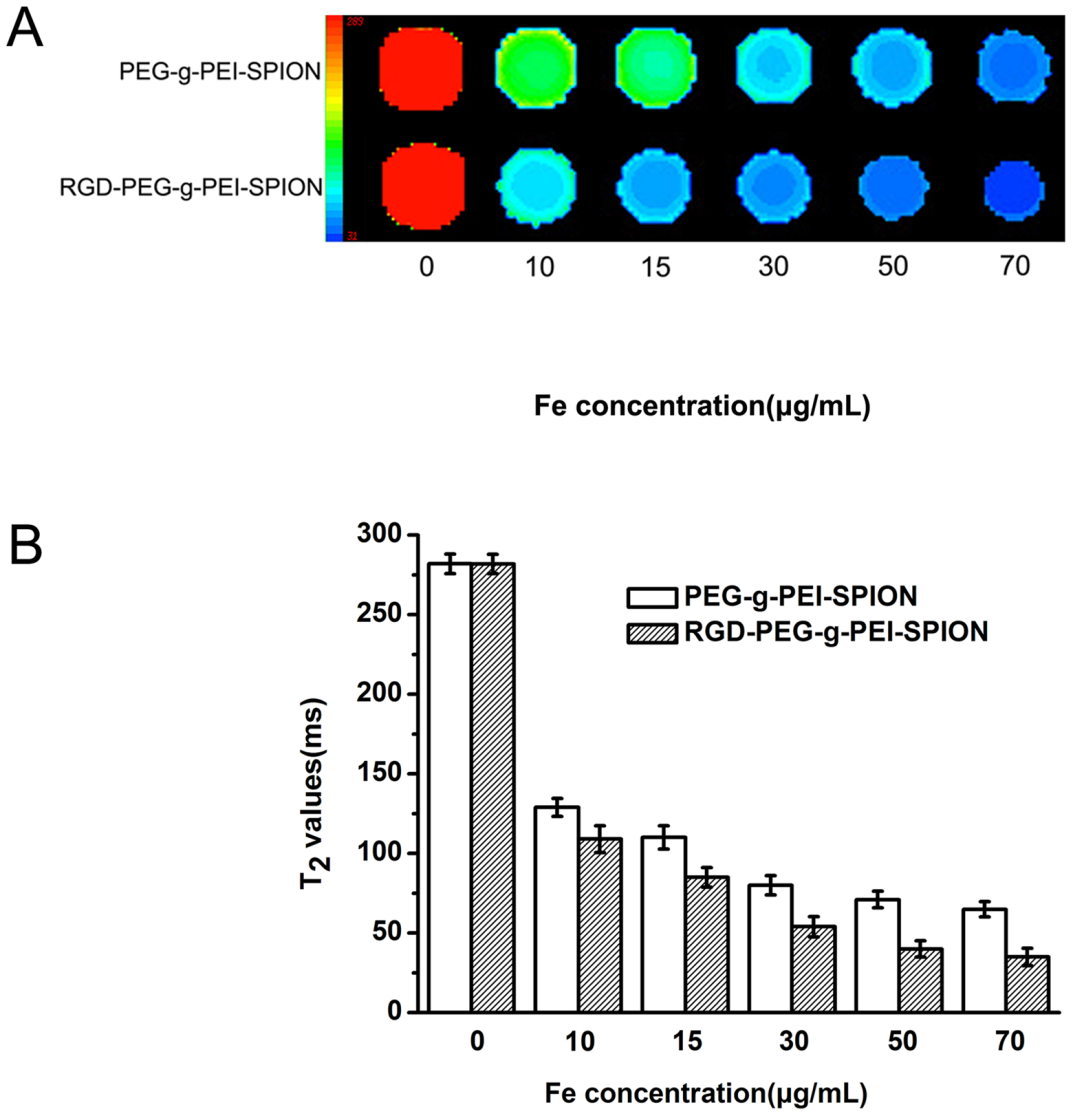
T2-mapping image of the Bel-7402 cells. (A) T_2_-mapping image of the Bel-7402 cells incubated with PEG-*g*-PEI-SPION or RGD-PEG-*g*-PEI-SPION at different Fe concentrations. (B) T_2_ values of the cells incubated with various complexes at different Fe concentration. (means±SD; n = 3).

MRI was performed to evaluate the abilities of RGD-PEG-*g*-PEI-SPION and PEG-*g*-PEI-SPION to target Bel-7402 cells. As shown in Figure S5A and B, the T_2_WI signal intensity of the Bel-7402 cells incubated with various complexes showed a significant decrease across the entire experimental Fe concentration range of 10 to 70 µg mL^−1^. At the same Fe concentration, the cells incubated with RGD-PEG-*g*-PEI-SPION exhibited a more remarkable darkening phenomenon and lower normalized MR signal intensity values than the cells incubated with PEG-*g*-PEI-SPION. These results indicate that RGD-PEG-*g*-PEI-SPION can be more efficiently taken up by the Bel-7402 cells, which over-express the integrin receptor. The T_2_-mapping images and T_2_ values were obtained for further analysis of the cell-targeting ability of the various complexes and to study the reason for the MR signal intensity reduction by the Fe. As shown in Figure 5 A and B, the colors of the T_2_-mapping image varied from red to blue, which represent the MR signal intensity, and the T_2_ value varied from high to low. With increasing Fe concentrations in the complexes, the trends of the T_2_-mapping images and T_2_ values exhibited by Bel-7402 cells incubated with the various complexes was in accordance with the aforementioned T_2_WI signal intensity. These results not only indicate that the RGD-modified complexes possess high transfection efficiency in Bel-7402 cells but also verify that the Fe reduces the T_2_WI signal intensity of the cells via shortening the T_2_ values at locations where the superparamagnetic iron oxide nanoparticles (SPION) accumulate.

### The Therapeutic Effect of Survivin Sirna Targeting *in vivo*

**Figure 6:**
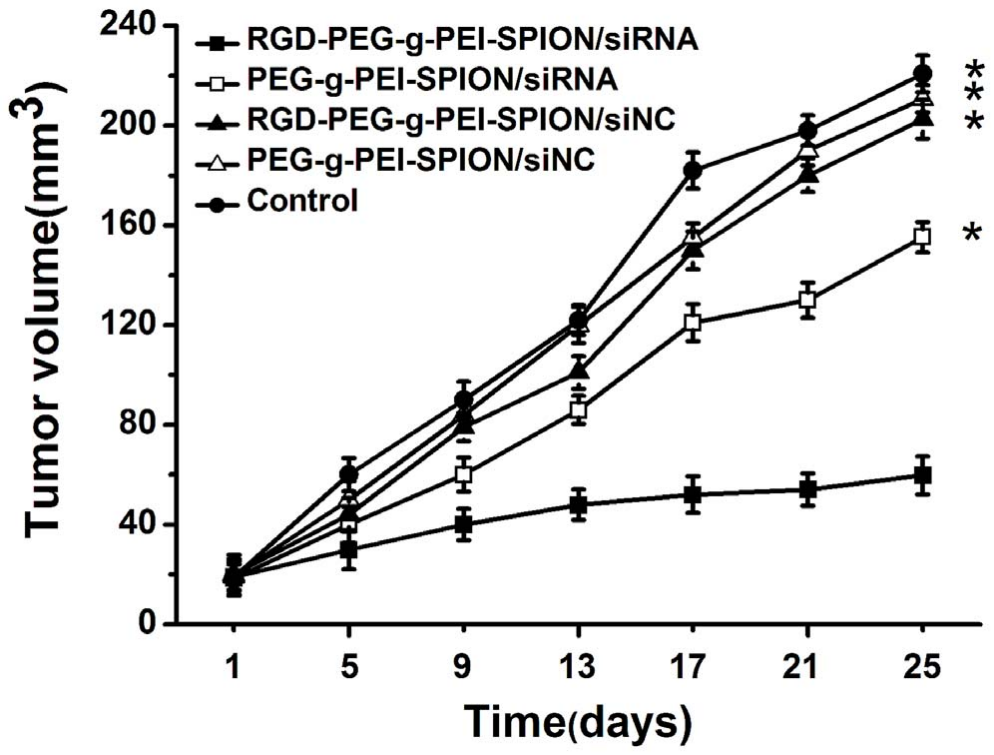
Tumor growth inhibition analysis. The tumor growth inhibition analysis in nude mice bearing Bel-7402 tumors after injection of various complexes formed at a N/P ratio of 10. (means±SD; n = 10; *p<0.05, compared with RGD-PEG-*g*-PEI-SPION/siRNA; Control: the mice injected with PBS).

The tumor growth inhibition study was performed on nude mice bearing Bel-7402 subcutaneous tumors via the systemic application of various complexes. As shown in Figure 6, 25 days after the first treatment, the tumor volumes of the mice injected with RGD-PEG-*g*-PEI-SPION/siRNA, PEG-*g*-PEI-SPION/siRNA, RGD-PEG-*g*-PEI-SPION/siNC, PEG-*g*-PEI-SPION/siNC and PBS were 59.8±7.6 mm^3^, 155.2±6.1 mm^3^, 202.5±7.8 mm^3^, 210.7±5.4 mm^3^, and 220.7±7.4 mm^3^, respectively. The mice injected with RGD-PEG-*g*-PEI-SPION/siRNA exhibited a better therapeutic effect (*p*<0.05) compared with the mice injected with the other complexes. These results not only demonstrate that Survivin siRNA can efficiently inhibit tumor growth but also further confirm that RGD-modified complexes possess a greater ability to deliver siRNA into Bel-7402 cells in vivo.

**Figure 7:**
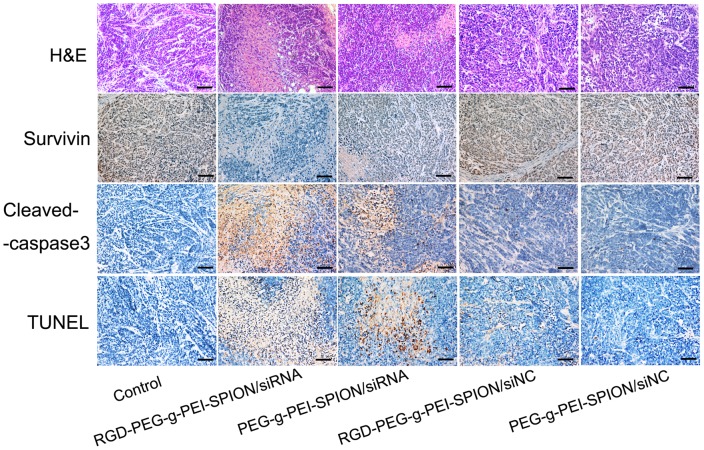
*Ex vivo* histological analyses of tumor tissue sections. The H&E staining, immunohistochemical and TUNEL analyses of tumor tissue sections from the mice treated with various complexes formed at a N/P ratio of 10. In the immunohistochemical assay, the brown stains indicate Survivin or cleaved caspase-3 protein. In the TUNEL assay, the brown stains indicate apoptotic cells (×200; scale bar:100 µm; Control: the mice injected with PBS).

The histological changes of tumors induced by different complexes were evaluated by an H&E staining assay. As shown in Figure 7, the nuclei were stained blue, whereas the extracellular matrix and cytoplasm were stained red. Among the mice injected with the various complexes, the tumor tissues excised from the mice injected with RGD-PEG-*g*-PEI-SPION/siRNA contained the fewest tumor cells and had the highest level of tumor necrosis. These results are in accordance with those of the tumor growth inhibition study.

In situ immunohistochemistry and TUNEL assays were performed to study the relationship between the Bel-7402 cell apoptosis and the Survivin protein in the tumor tissues of mice injected with various complexes. As shown in Figure 7, in the in situ immunohistochemical study, the cell nuclei were stained blue, and the brown stains represented the Survivin or cleaved caspase-3 protein expressed in the tumor tissue. In comparison with the expression levels of the Survivin and cleaved caspase-3 protein in the tumor tissues from the mice injected with the other complexes, the tumor tissues from the mice injected with RGD-PEG-*g*-PEI-SPION/siRNA exhibited the lowest level of the Survivin protein and the highest level of the cleaved caspase-3 protein with regard to the distribution range and intensity of protein expression. In the situ TUNEL study, the cells whose nuclei were stained brown represented the apoptotic cells. The tumor tissues of the mice injected with RGD-PEG-*g*-PEI-SPION/siRNA exhibited a greater number of apoptotic cells than those from the mice injected with the other complexes.

The immunohistologic analysis of the intratumoral microvascular density (MVD) was used to assess angiogenesis. As shown in Figure S6, the blood vessels were highlighted by staining endothelial cells for CD34 (brown). The measurements of MVD were performed independently by two pathologists (with 8 years of working experience in MVD) who were blinded to the tumor treatment according to the method described by Weidner et al. [16] The tumor mean MVD of the mice injected with RGD-PEG-*g*-PEI-SPION/siRNA, PEG-*g*-PEI-SPION/siRNA, RGD-PEG-*g*-PEI-SPION/siNC, PEG-*g*-PEI-SPION/siNC and PBS were 29.9±2.1 per 400×field, 30.5±1.5 per 400×field, 31.4±2.8 per 400×field, 30.9±1.7 per 400×field, and 31.7±1.8 per 400×field, respectively. NO significant difference was observed in MVD among the various treatment groups. These results indicated that the RGD-modified complexes has no significant effect on tumor angiogenesis.

### *in vivo* Mr Imaging

**Figure 8:**
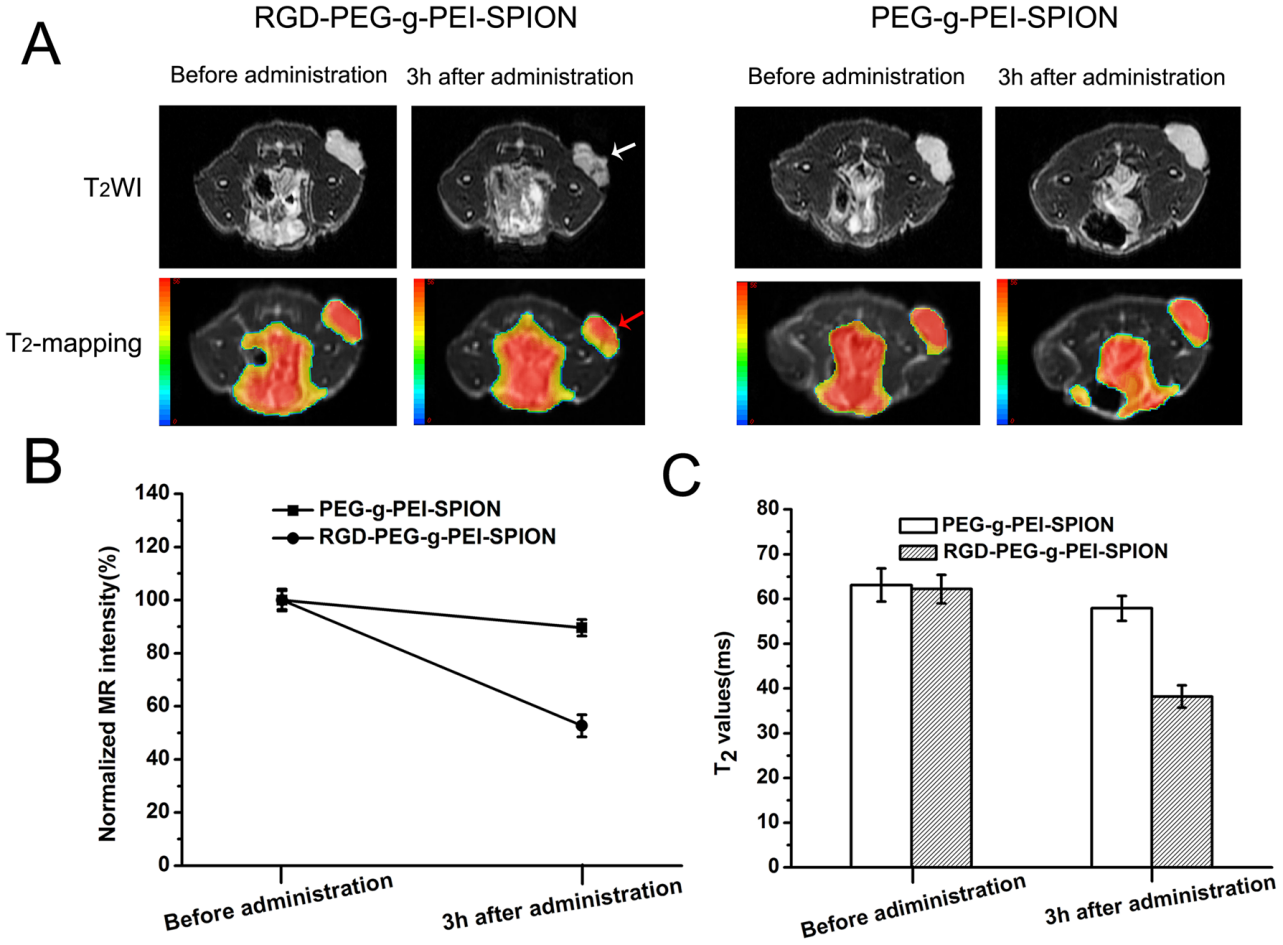
*In vivo* tumor targeting evaluation. (A) T_2_WI and T_2_-mapping images of mice bearing Bel-7402 subcutaneous tumors before and after tail vein administration of RGD-PEG-*g*-PEI-SPION or PEG-*g*-PEI-SPION. (B) The normalized MR signal intensity of the tumors before and after tail vein administration of the various complexes. (C) T_2_ values of the tumors before and after tail vein administration of the various complexes. (means±SD; n = 5).

In vivo Bel-7402 tumor MR imaging was performed to further investigate the tumor targeting abilities of RGD-PEG-*g*-PEI-SPION and PEG-*g*-PEI-SPION. The various complexes were injected via tail veins into nude mice bearing Bel-7402 tumors. As shown in Figure 8A, all the tumors presented high signal intensity on T_2_WI before administration of the various complexes. Three hours after the administration of RGD-PEG-*g*-PEI-SPION, the Bel-7402 tumors exhibited an obviously non-uniform low-intensity signal on T_2_WI (white arrow), and the red color range in the tumor decreased significantly in the T_2_-mapping image (red arrow). Consistent with this result, the normalized MR signal intensity value of the tumor decreased to 52.7%±4.2% (Figure 8B), and the T_2_ value of the tumor decreased from 62.2±3.2 ms to 38.2±2.5 ms (Figure 8C). In contrast, 3 h after the administration of PEG-*g*-PEI-SPION, the Bel-7402 tumors did not exhibit a distinct change in either the signal intensity on T_2_WI or the range of red color in the T_2_-mapping image (Figure 8A). The normalized MR signal intensity value of the tumor only decreased to 89.6%±3.1% (Figure 8B), and the T_2_ value of the tumor only decreased from 63.1±3.7 ms to 57.9±2.8 ms (Figure 8C).

To further verify the enhanced accumulation of the RGD-PEG-*g*-PEI-SPION in the tumor, Prussian blue staining of the tumor tissue was performed. As shown in Figure S7, the tumor tissue sections excised from the mice injected with RGD-PEG-*g*-PEI-SPION showed numerous blue dots (black arrow), whereas the tumor tissue sections excised from the mice injected with PEG-*g*-PEI-SPION showed few blue dots. All of these results further indicate that RGD-PEG-*g*-PEI-SPION possesses Bel-7402 tumor-targeting ability greater than that of PEG-*g*-PEI-SPION.

## Discussion

In our recent report, an MRI-visible non-viral vector was successfully conjugated with a specific targeting ligand. Our results showed that this vector could effectively deliver a therapeutic gene into neuroblastoma cells, which resulted in a satisfactory antitumor effect and a tumor MR imaging effect [14].This inspiring success led us to further explore targeted gene therapy and MR localization of hepatocellular carcinoma.

Currently, a potential approach for targeting specific mRNA, RNAi, is being used for gene silencing in various diseases, especially tumor therapy. As an anti-apoptotic protein, Survivin can inhibit apoptosis via targeting the intrinsic pathway of cell death, such as controlling spindle formation and proper kinetochore attachment during cell division. Survivin also can maintain endothelial cell viability during the proliferative and remodeling phases of angiogenesis [8]. Survivin is over-expressed in hepatocellular carcinoma cells and promotes HCC progression via upregulation of the *survivin* proto-oncogene [17], [18]. However, survivin is rarely expressed in terminally differentiated adult tissues. Therefore, survivin is regarded as a promising hepatocellular carcinoma therapeutic target. Using RNAi technology, we utilized the Survivin siRNA to suppress the expression of the Survivin protein via silencing the Survivin mRNA in Bel-7402 cells and, thereby, induced tumor cell apoptosis. For efficient tumor therapy, the siRNA should be stable, efficiently delivered into the target tissue, and easily taken up by the tumor cells [19]. Previously, we conjugated a single-chain antibody directed against GD2 to a non-viral vector (PEG-*g*-PEI-SPION). The modified vector successfully co-delivered more therapeutic gene and SPION into neuroblastoma cells and achieved better antitumor and tumor MR imaging effects. These results indicate that the targeted non-viral vector could significantly improve the transfection efficiency in tumors in comparison with the non-targeted non-viral vector [14]. The integrin receptors α_ν_β_3_ and α_ν_β_5_ are overexpressed on actively proliferating endothelial cells and malignant tumor cells within solid tumors. The surface of HCC cells abounds with these specific integrin receptors, whereas α_ν_β_3_ and α_ν_β_5_ are rarely expressed in normal hepatocytes. These markers can be specifically recognized by RGD [20]. Thus, based on our previous study, we employed the RGD peptide as a hepatocellular carcinoma cell-targeting ligand and synthesized an RGD-modified non-viral nanocarrier, RGD-PEG-*g*-PEI-SPION.

The results of agarose gel electrophoresis showed that both RGD-PEG-*g*-PEI-SPION and PEG-*g*-PEI-SPION could complex entirely with the siRNA at a N/P ratio of 2.5, which demonstrates that the negative charge of the siRNA is neutralized by the positively charged vector and makes it possible to deliver the siRNA into cells. Furthermore, there was no significant distinction in the ability to condense siRNA between the RGD-PEG-*g*-PEI-SPION and PEG-*g*-PEI-SPION nanocarriers, which indicates that the RGD did not influence the ability of the vectors to condense siRNA. The seemly weak positive zeta potential [21] and small particle size [22] are known to favor the cellular uptake of nanoparticles. The seemly weak positive charge facilitates the adhesion of nanoparticles to cells via electrostatic interactions; however, an exorbitant positive charge will result in excessive amounts of cations attaching to the cell membrane and will lead to high cytotoxicity. A small particle size contributes to endocytosis of the nanoparticle. In the present study, at a N/P ratio of 10, the particle size of RGD-PEG-*g*-PEI-SPION/siRNA tended to be stable and reached 85.1±5.7 nM in diameter. Under the same conditions, the zeta potential of RGD-PEG-*g*-PEI-SPION/siRNA also reached an ideal value. These results imply that the size and zeta potential of the complexes at a N/P ratio of 10 are suitable for cell transfection.

The ability of RGD-PEG-*g*-PEI-SPION to deliver siRNA into Bel-7402 cells in vitro was evaluated by flow cytometry, fluorescence microscopy and laser confocal microscopy. According to the quantitative flow cytometric analysis, the percentage of FITC-positive cells from among the cells incubated with all the various complexes reached a peak at a N/P ratio of 10. In addition, the percentage of FITC-positive cells increased by approximately 2-fold when the cells were incubated with RGD-PEG-*g*-PEI-SPION/siRNA compared with the cells incubated with PEG-*g*-PEI-SPION/siRNA or RGD-PEG-*g*-PEI-SPION/siRNA in the presence of free RGD. The fluorescence images of the cells incubated with the RGD-PEG-*g*-PEI-SPION/siRNA exhibited a stronger green fluorescence intensity at a N/P ratio of 10 compared with that of the cells incubated with the other complexes. Furthermore, the laser confocal images of the cells incubated with RGD-PEG-*g*-PEI-SPION/siRNA also showed stronger RGD-PEG-*g*-PEI-SPION (red) and siRNA (green) fluorescence compared with that of the cells incubated with the other complexes. These results all demonstrate that the RGD-mediated non-viral vector, RGD-PEG-*g*-PEI-SPION, can deliver more siRNA into Bel-7402 cells with high efficiency than can PEG-*g*-PEI-SPION. The MTT assay showed that the cytotoxicity of the various complexes all increased concurrently with the increasing N/P ratios. At a N/P ratio of 10, the viability of the cells incubated with RGD-PEG-*g*-PEI-SPION/siNC and PEG-*g*-PEI-SPION/siNC was greater than 90%. No significant difference in cytotoxicity at different N/P ratios between RGD-PEG-*g*-PEI-SPION/siNC and PEG-*g*-PEI-SPION/siNC was observed, which indicates that the RGD had no obvious cytotoxicity to Bel-7402 cells. However, at the same N/P ratio, the RGD-PEG-*g*-PEI-SPION/siRNA exhibited a cytotoxicity higher than that of the other complexes, which strongly demonstrates that the binding of RGD could enhance the ability of the vector to deliver Survivin siRNA into Bel-7402 cells and achieve a better therapeutic effect. The ability of RGD-PEG-*g*-PEI-SPION/siRNA to suppress the expression of the *Survivin* gene in Bel-7402 cells was evaluated at both the mRNA and the protein levels. The RT-PCR assay and the western blotting assay revealed that the cells incubated with RGD-PEG-*g*-PEI-SPION/siRNA exhibited a lower Survivin mRNA expression level and a weaker Survivin protein band comparison with those of the cells incubated with the other complexes. At the same time, the cells treated with RGD-PEG-*g*-PEI-SPION/siRNA expressed the strongest cleaved caspase-3 protein band, which is a good indicator of cell apoptosis. The subsequent TUNEL assay in vitro further verified that the Bel-7402 cells incubated with RGD-PEG-*g*-PEI- SPION/siRNA showed the highest level of cell apoptosis. In the tumor growth inhibition study, the mice injected with RGD-PEG-*g*-PEI-SPION/siRNA obtained the best therapeutic effect compared to the mice injected with the other complexes. The H&E staining assay showed that the tumor tissues excised from the mice injected with RGD-PEG-*g*-PEI-SPION/siRNA exhibited the fewest number of tumor cells and the highest level of tumor necrosis. The in situ immunohistochemistry and TUNEL assays performed in the in vivo experiments further confirmed that the Survivin siRNA could be effectively delivered into Bel-7402 cells by the RGD-modified nanocarrier. These assays also indicated that the delivery of the Survivin siRNA resulted in downregulation of the *Survivin* gene expression and the upregulation of cleaved caspase-3 expression, which led to a higher level of cellular apoptosis than did the non-targeted therapy in vivo.

Because SPION can produce a low signal intensity on T_2_WI, the Bel-7402 tumor-targeting ability of RGD-PEG-*g*-PEI-SPION can be noninvasively monitored by an MRI scan in vitro and in vivo. Under the same conditions, the normalized MR signal intensity values and T_2_ values of the cells incubated with RGD-PEG-*g*-PEI-SPION were always lower than those of the cells incubated with PEG-*g*-PEI-SPION. After administration of RGD-PEG-*g*-PEI-SPION, the Bel-7402 tumors exhibited an obvious decrease in both normalized MR signal intensity values and T_2_ values, whereas after administration of PEG-*g*-PEI-SPION, the Bel-7402 tumors showed no significant change in normalized MR signal intensity values and T_2_ values. The Prussian blue staining confirmed that the tumor tissue sections excised from the mice injected with RGD-PEG-*g*-PEI-SPION showed more SPION accumulation. These results are also consistent with the finding that RGD-modified complexes possess higher transfection efficiency in Bel-7402 cells. As an iron-containing compound, the RGD-modified complexes are selectively taken up and metabolized by the reticuloendothelial system (RES) after injected via the tail vein, mostly in the liver and spleen [23], [24]. In the present study, the RGD-modified targeting delivery is an strategy for enhancing transfection efficiency and meanwhile reducing toxicity and other side effects because it enables nanoparticles to specifically act on tumor cells.

## Conclusion

We synthesized an RGD-modified MRI-visible non-viral vector for specifically delivering Survivin siRNA to Bel-7402 cells and for sensitively detecting tumor cells by MRI. Both in vitro and in vivo studies demonstrated that this non-viral vector efficiently delivered Survivin siRNA to HCC cells, which resulted in obvious tumor cell apoptosis and the significant inhibition of Bel-7402 tumor growth. The Bel-7402 tumor-targeting ability of this non-viral vector was sufficiently visualized by an MRI scan. Our results revealed that the RGD-modified MRI-visible non-viral vector may be an effective platform for HCC-targeted siRNA therpay and MR imaging that deserves further investigation.

## Supporting Information

Text S1 **Synthesis of RGD-PEG-** ***g*** **-PEI-SPION in detail.**
(DOC)

Figure S1 **Determination of siRNA complexation by gel retardation assay.** Agarose gel electrophoretic mobility of siRNA after complexing with RGD-PEG-*g*-PEI-SPION and PEG-*g*-PEI-SPION at various N/P ratios.
(TIF)

Figure S2 **Zeta potential and particle size measurements.** (A) Zeta potential and particle size of RGD-PEG-*g*-PEI-SPION non-complexed or complexed with siRNA at different N/P ratios. (B) Zeta potential and particle size of PEG-*g*-PEI-SPION non-complexed or complexed with siRNA at different N/P ratios. (mean±SD, n = 3).
(TIF)

Figure S3 **Expression of ανβ3 in the Bel-7402 cells.** (A) The immunocytochemistry findings of α_ν_β_3_ in the Bel-7402 cells. The brown areas indicate positive α_ν_β_3_ staining. (B) Negative control group added PBS instead of antibody. (×400; scale bar: 50 µm).
(TIF)

Figure S4 **Flow cytometry analysis and fluorescence images.** (A) Quantitative analysis by flow cytometry of FITC-positive cells incubated with RGD-PEG-*g*-PEI-SPION/siRNA, PEG-*g*-PEI-SPION/siRNA or RGD-PEG-*g*-PEI-SPION/siRNA in the presence of free RGD at a N/P ratio of 10. (B) The fluorescence and bright-field microscope images of cells incubated with various complexes at a N/P ratio of 10. (×200; scale bar: 100 µm).
(TIF)

Figure S5 **T2-weighted image of the Bel-7402 cells.** (A) T_2_WI of the Bel-7402 cells incubated with PEG-*g*-PEI-SPION or RGD-PEG-*g*-PEI-SPION at different Fe concentrations. (B) The normalized MR signal intensity of the cells incubated with various complexes at different Fe concentrations.
(TIF)

Figure S6 **Immunohistochemical analyses of tumor angiogenesis.** (A)The immunohistochemical analyses of tumor tissue sections from the mice treated with various complexes formed at a N/P ratio of 10. The brown areas indicate positive CD34 staining of endothelial cells. (×400; scale bar: 50 µm). (B) MVD assessed from the various treatment groups. (means±SD; n = 3; Control: the mice injected with PBS).
(TIF)

Figure S7 **Prussian blue staining was performed on tumor tissues.** Prussian blue staining of tumor tissue sections excised from the mice after tail vein administration of RGD-PEG-*g*-PEI-SPION or PEG-*g*-PEI-SPION. (×400; scale bar: 50 µm.).
(TIF)
