# Supplementary material for: An RGD-Modified MRI-Visible Polymeric Vector for Targeted siRNA Delivery to Hepatocellular Carcinoma in Nude Mice
Source: PLoS One. 2013 Jun 7;8(6):e66416. doi: 10.1371/journal.pone.0066416 (PMC3676333; doi:10.1371/journal.pone.0066416)
Supplement: Text S1 — Synthesis of RGD-PEG- g -PEI-SPION in detail. (DOC) [file pone.0066416.s001.doc]

**Synthesis of RGD-PEG-*g*-PEI-SPION**

1. Synthesis of PEG-g-PEI-SPION

Fe_3_O_4_ nanocrystals were synthesized according to a method reported by Sun et al [Sun S, Zeng H, Robinson DB, Raoux S, Rice PM, et al. (2004) Monodisperse MFe_2_O_4_ (M = Fe, Co, Mn) nanoparticles. J. Am. Chem. Soc. 126: 273-279]. Briefly, iron(III) acetylacetonate (2 mmol), 1, 2-hexadecanediol (10 mmol), oleic acid (6 mmol), oleylamine (6 mmol), and benzyl ether (20 ml) were mixed in a reaction bottle by magnetic stirring under a nitrogen flow. The mixture was heated to 200℃ and reacted for 2 h, and finally refluxed at 300℃ for another 1 h. The black mixture was cooled to 37℃, precipitated into ethanol and then centrifuged (12 000 rpm, 10 min) to remove the solvent. The black-brown nanoparticles, Fe_3_O_4_ nanoparticles measuring 6 nm, were dissolved in hexane for storage prior to use.

PEG-g-PEI-SPION was synthesized as our reported work [Chen G, Chen W, Wu Z, Yuan R, Li H, et al. (2009) MRI-visible polymeric vector bearing CD3 single chain antibody for gene delivery to T cells for immunosuppression. Biomaterials 30:1962-1970]. In brief, PEG- g-PEI was synthesized by conjugating CDI-activated PEG to PEI. PEG-g-PEI-SPION was synthesized by “ligand” exchange method .

2. Synthesis of RGD-PEG-COOH

RGD-PEG-COOH was synthesized as our reported work [ Li L, Yang J, Wang WW, Yao YC, Fang SH, et al. (2012) Pigment epithelium-derived factor gene loaded in cRGD - PEG-PEI suppresses colorectal cancer growth by targeting endothelial cells. Int J Pharm 438: 1-10]. In brief, (i) allyl-PEG-OH. Tetrahydrofuran (THF; 40 ml) was added into naphthalene (2 g). After naphthalene dissolving, metallic potassium (2 g) was added and mixed for 30 min. 18-crown-6-ether (1.4 g) was dissolved in 40 ml anhydrous THF. After naphthalene potassium solution was mixed with propylene alcohol solution (0.28 ml), the mixture was added 18-crown-6-ether solution and stirred for 30 min (pH 4.5). The reaction flask was put in the icy saline water and blew with oxacyclopropane, and then gone on reacting for 3 days. After being precipitated and washed with anhydrous ether, the allyl-PEG-OH was synthesized. (ii) NH_2_-PEG-OH. After allyl-PEG-OH(4000 Da; 3.0 g) was dissolved in DMF solution (10 ml), the solution was reacted with 2-mercaptoethylamine hydrochloride （1.3 g） and azodiisobutyronitrile (AIBN; 0.1 g) for 1 day (pH 10.0), subsequently extracted with chloroform and collected with organic phase. Then the organic phase was dried by anhydrous sodium sulfate and precipitated in anhydrous ether. The NH_2_-PEG-OH was filtrated from anhydrous ether. (iii) mal-PEG-OH. After NH_2_-PEG-OH(4000 Da; 2 g) was dissolved in saturated sodium bicarbonate solution below zero, N-methoxycarbonylmaleimide (0.4 g) and 15% NaCl were added in sequence (pH 3.0). The mixture was extracted with dichloromethane, and then the extraction was dehydrated and filtrated. After the reactant product was precipitated and dried at 37 ℃, the mal-PEG-OH was obtained. (iv) mal-PEG-COOH. Mal-PEG-COOH was manufactured with mal-PEG-OH (0.3 g), trichloromethane (20 ml) and butanedioic anhydride (0.05 g). (v) RGD–PEG-COOH. [Nasongkla N, Shuai X, Ai H, Weinberg BD, Pink J, et al. (2004) RGD-functionalized polymer micelles for targeted doxorubicin delivery. Angew chem Int Ed Engl. 43: 6323-6327.] RGD (1 mg) was dissolved in 500 μl of 4-(2-hydroxyethyl)-1-piperazineethanesulfonic acid/ ethylene diamine tetraacetic acid (HEPES/EDTA) solution (0.5 M, pH 8.0). Hydroxylamine (200 μl) was mixed with HEPES/EDTA (500 μl) and was then added to the RGD solution. After incubation at 37°C for 90 min, 300 μg of heterofunctional PEG terminated with carboxyl and maleimide groups (MAL-PEG-COOH) dissolved in 200 μl of 0.5 M HEPES/EDTA was added to the RGD solution and incubated at 4°C overnight. The resulting solution was dialyzed by membrane dialysis (molecular weight cutoff: 1000 Da) for 24 h and lyophilized to obtain 200 μg white powder of RGD–PEG-COOH.

1. Synthesis of RGD-PEG-PEI-SPION.

100 μg RGD-PEG-COOH was dissolved in 1 ml water and reacted with 10 μg EDC and 10 μg NHS. 10 min later, PEG-g-PEI-SPION (100 μl, 1 μg/μl) was added and reacted overnight at 4℃. The resulting solution was purified by ultrafiltration in an Amicon cell (regenerated cellulose membrane, MWCO=10000) for 3 times, washing with PBS (each 500 μL containing 10 μL of 0.5 M EDTA solution, pH 7.4 ). The final product, RGD-PEG-*g*-PEI-SPION, was then recovered.
