# Supplementary figures and images for: An RGD-Modified MRI-Visible Polymeric Vector for Targeted siRNA Delivery to Hepatocellular Carcinoma in Nude Mice
Source: PLoS One. 2013 Jun 7;8(6):e66416. doi: 10.1371/journal.pone.0066416 (PMC3676333; doi:10.1371/journal.pone.0066416)

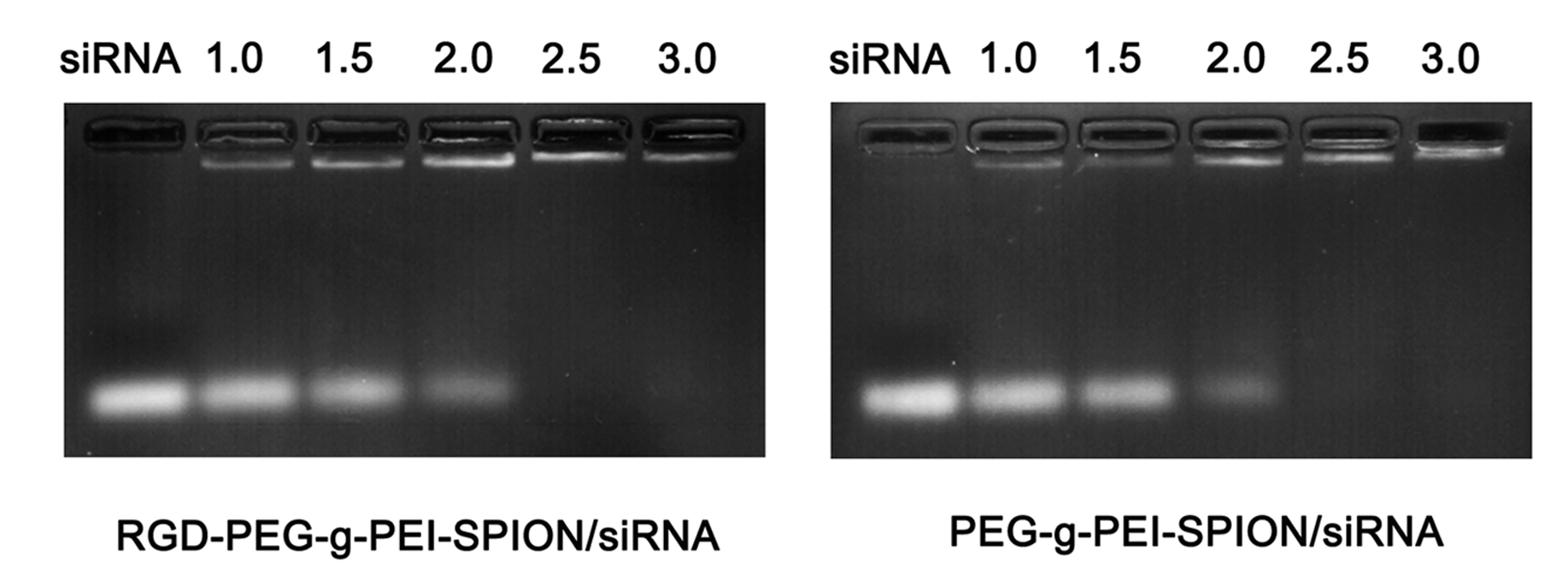

Supplement: Figure S1 — Determination of siRNA complexation by gel retardation assay. Agarose gel electrophoretic mobility of siRNA after complexing with RGD-PEG-g-PEI-SPION and PEG-g-PEI-SPION at various N/P ratios. (TIF) [file pone.0066416.s002.tif]

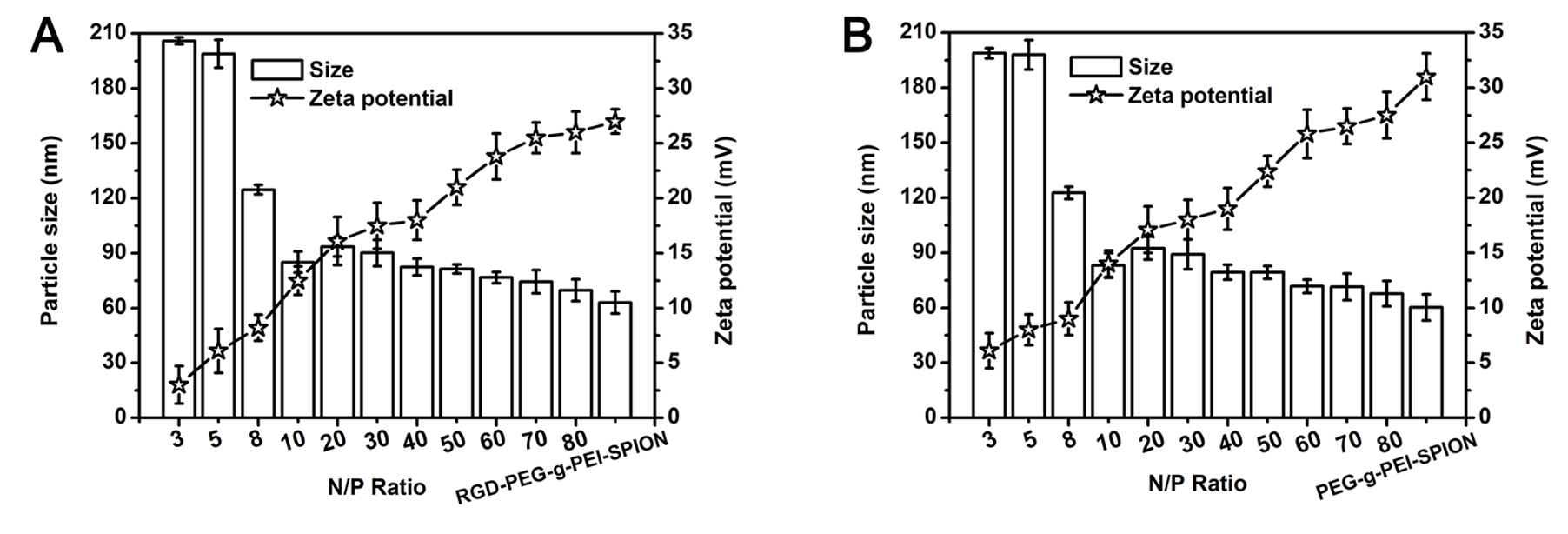

Supplement: Figure S2 — Zeta potential and particle size measurements. (A) Zeta potential and particle size of RGD-PEG-g-PEI-SPION non-complexed or complexed with siRNA at different N/P ratios. (B) Zeta potential and particle size of PEG-g-PEI-SPION non-complexed or complexed with siRNA at different N/P ratios. (mean±SD, n = 3). (TIF) [file pone.0066416.s003.tif]

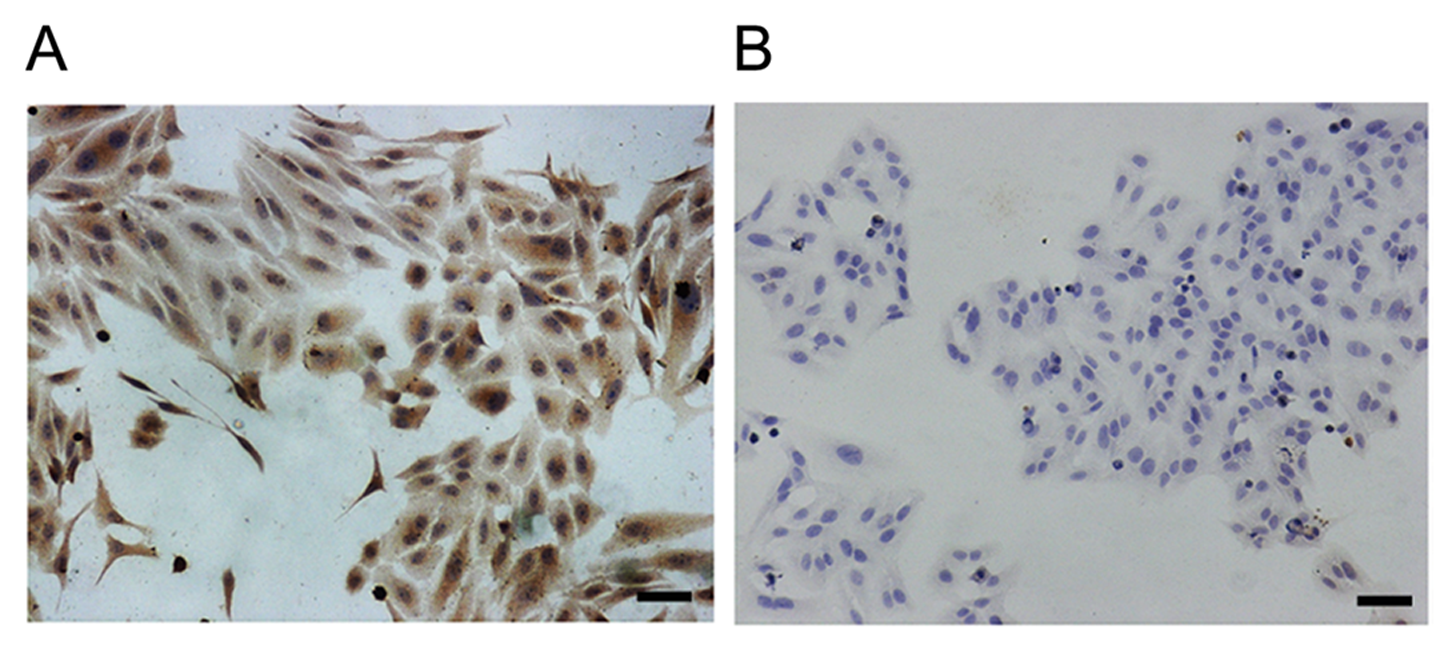

Supplement: Figure S3 — Expression of ανβ3 in the Bel-7402 cells. (A) The immunocytochemistry findings of ανβ3 in the Bel-7402 cells. The brown areas indicate positive ανβ3 staining. (B) Negative control group added PBS instead of antibody. (×400; scale bar: 50 µm). (TIF) [file pone.0066416.s004.tif]

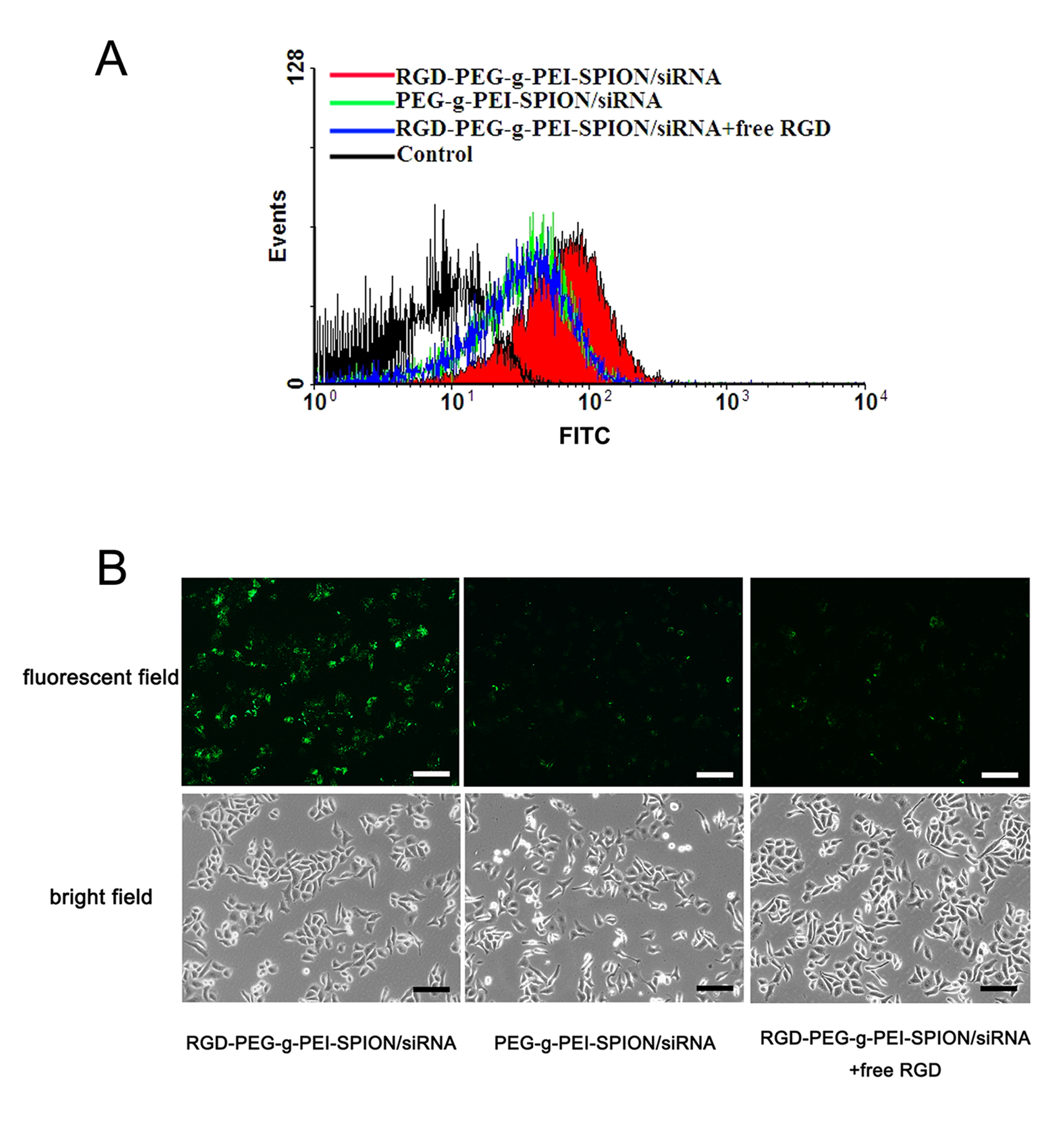

Supplement: Figure S4 — Flow cytometry analysis and fluorescence images. (A) Quantitative analysis by flow cytometry of FITC-positive cells incubated with RGD-PEG-g-PEI-SPION/siRNA, PEG-g-PEI-SPION/siRNA or RGD-PEG-g-PEI-SPION/siRNA in the presence of free RGD at a N/P ratio of 10. (B) The fluorescence and bright-field microscope images of cells incubated with various complexes at a N/P ratio of 10. (×200; scale bar: 100 µm). (TIF) [file pone.0066416.s005.tif]

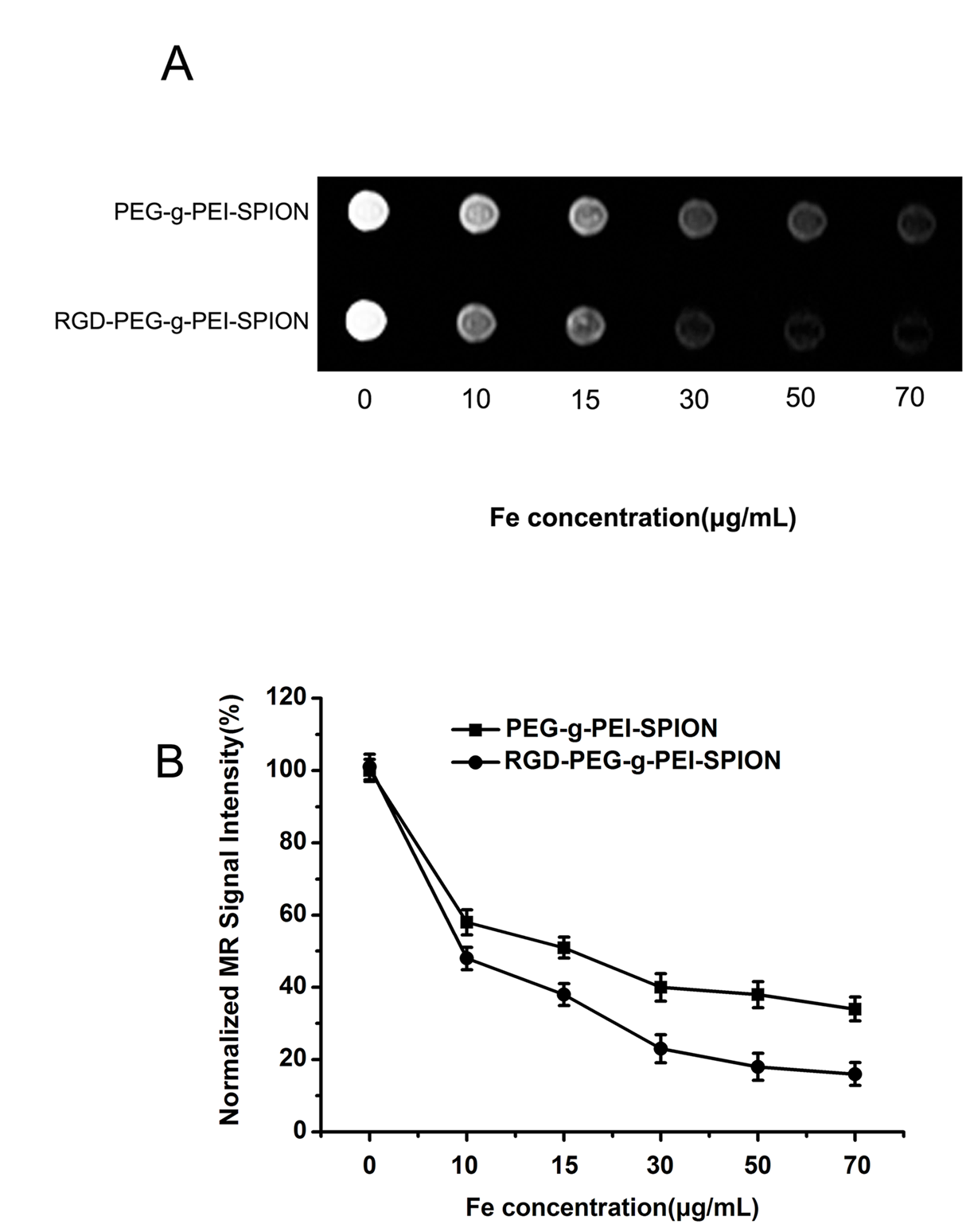

Supplement: Figure S5 — T2-weighted image of the Bel-7402 cells. (A) T2WI of the Bel-7402 cells incubated with PEG-g-PEI-SPION or RGD-PEG-g-PEI-SPION at different Fe concentrations. (B) The normalized MR signal intensity of the cells incubated with various complexes at different Fe concentrations. (TIF) [file pone.0066416.s006.tif]

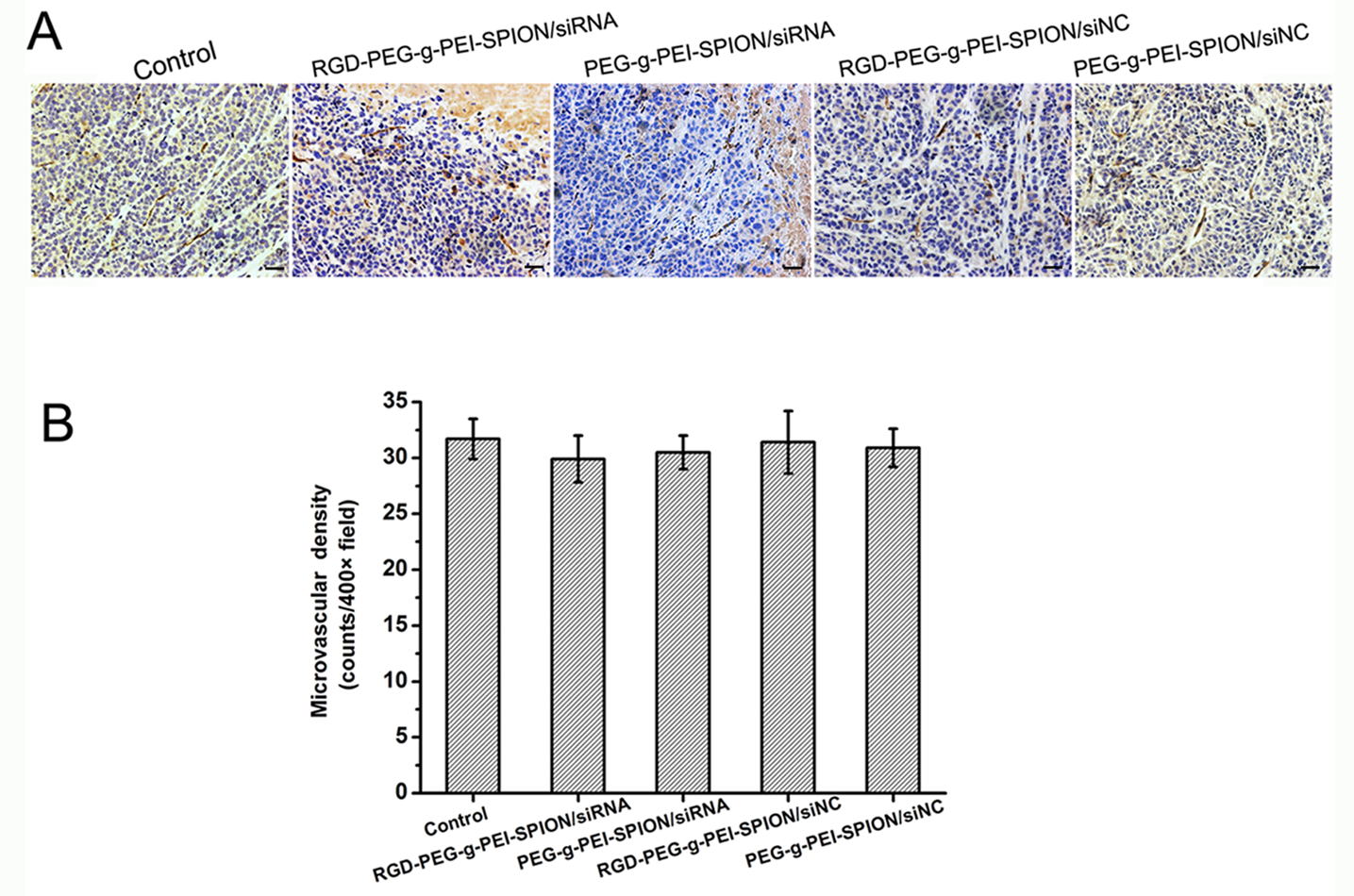

Supplement: Figure S6 — Immunohistochemical analyses of tumor angiogenesis. (A)The immunohistochemical analyses of tumor tissue sections from the mice treated with various complexes formed at a N/P ratio of 10. The brown areas indicate positive CD34 staining of endothelial cells. (×400; scale bar: 50 µm). (B) MVD assessed from the various treatment groups. (means±SD; n = 3; Control: the mice injected with PBS). (TIF) [file pone.0066416.s007.tif]

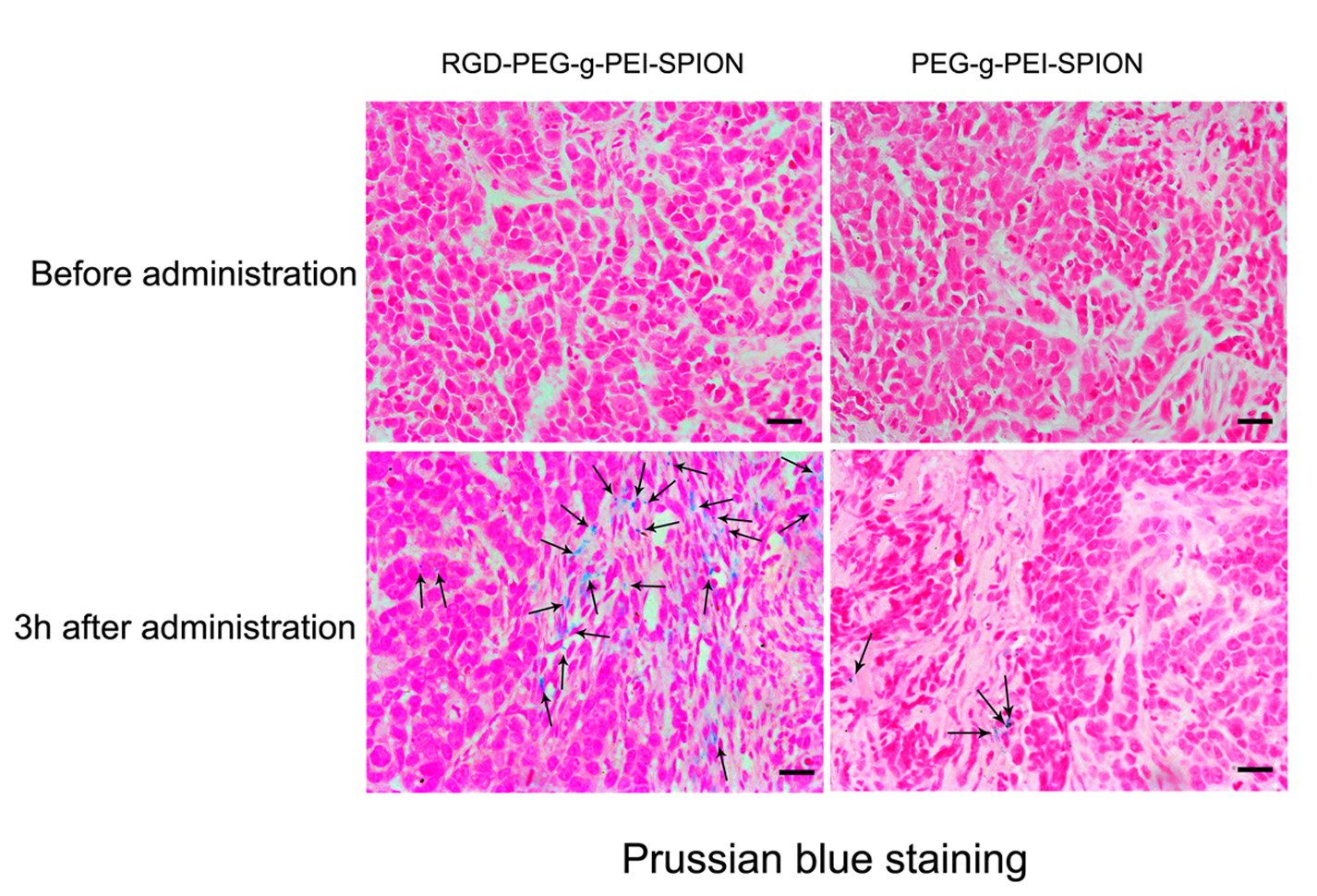

Supplement: Figure S7 — Prussian blue staining was performed on tumor tissues. Prussian blue staining of tumor tissue sections excised from the mice after tail vein administration of RGD-PEG-g-PEI-SPION or PEG-g-PEI-SPION. (×400; scale bar: 50 µm.). (TIF) [file pone.0066416.s008.tif]
